# Association between leukotriene receptor antagonists and neuropsychiatric disorders: a systematic review and meta-analysis

**DOI:** 10.3389/fphar.2026.1787744

**Published:** 2026-04-30

**Authors:** Jiawen Xian, Shipeng Zhang, Yujie Zhang, Ke Li, Qinxiu Zhang

**Affiliations:** 1 Chengdu University of Traditional Chinese Medicine, Chengdu, China; 2 Shi’s Medicine Center of Orthopedics and Traumatology, Shuguang Hospital Affiliated to Shanghai University of TCM, Shanghai, China; 3 School of Medical and Life Sciences, Chengdu University of Traditional Chinese Medicine, Chengdu, China; 4 World Health Organization Collaborating Centre (WHOCC), CHN-56, Chengdu, China

**Keywords:** adverse drug reactions, leukotriene receptor antagonists, meta-analysis, montelukast, neuropsychiatric disorders

## Abstract

**Objective:**

This study conducted a meta-analysis of previous research to comprehensively evaluate the association between leukotriene receptor antagonists (LTRAs) and the risk of neuropsychiatric disorders, with a specific focus on examining potential variations in this association across different age groups.

**Methods:**

A comprehensive search was conducted across multiple databases, including PubMed, Embase, Web of Science and the Cochrane Library, from their inception until 28 April 2025, with no language restrictions applied. The methodological quality of the included studies was assessed using the Newcastle-Ottawa Scale (NOS). Data analysis was performed using R (version 4.2.2), with results expressed as relative risk (RR) and 95% confidence intervals (CI). Sensitivity analysis was carried out to verify the robustness of the findings. Heterogeneity was quantified using the I^2^ statistic, while publication bias was evaluated through Egger’s test and visual inspection of funnel plots.

**Results:**

A meta-analysis of the 21 included studies revealed a borderline non-significant association between LTRA use and neuropsychiatric risk in the overall population (RR = 1.11, 95% CI: 0.98–1.26). However, subgroup analysis indicated a statistically significant age-dependent disparity in this risk. Specifically, no significant increase in overall risk was observed in the pediatric population (RR = 1.12, 95% CI: 0.90–1.39). In contrast, a statistically significant positive association was identified in the adult population (RR = 1.30, 95% CI: 1.08–1.56).

**Conclusion:**

Our findings indicated that LTRA use was associated with a favorable neuropsychiatric safety profile in the pediatric population, but was linked to an increased risk in adults. These results highlighted the need for age-specific risk assessment in clinical management.

**Systematic Review Registration:**

CRD420251042206.

## Introduction

Asthma was a prevalent chronic airway disease that affected approximately 330 million people worldwide (Chen et al., 2016; To et al., 2012). According to the Global Initiative for Asthma (GINA), its management typically involved long-term maintenance therapy combined with as-needed short-term treatment to alleviate acute symptoms (Boulet et al., 2015). Leukotriene receptor antagonists (LTRAs)—including montelukast, zafirlukast, and pranlukast—were widely used for the treatment of asthma and allergic rhinitis (Balzano et al., 2002). Montelukast, in particular, served as a foundational therapy due to its efficacy in reducing airway inflammation and bronchoconstriction (Buccellati et al., 2002). By antagonizing cysteinyl leukotriene receptors, this class of agents inhibited the airway inflammatory response mediated by leukotrienes and their derivatives (Boulet et al., 2015) and was also suggested to exert certain neuroprotective effects (Yagami et al., 2013).

Although the respiratory benefits of LTRAs were well-established, concerns regarding their neuropsychiatric safety profile had persisted since 2008 (Wallerstedt et al., 2009). These concerns initially emerged from post-marketing surveillance and spontaneous adverse event reports, which suggested a potential association between LTRAs and adverse neuropsychiatric events (Marchand et al., 2013; Philip et al., 2009; Schumock et al., 2011). On 4 March 2020, the U.S. Food and Drug Administration (FDA) issued a drug safety communication, imposing a Boxed Warning—the agency’s most stringent safety alert concerning serious neuropsychiatric events such as agitation, depression, sleep disturbances, and suicidal thoughts and behaviors—on the asthma and allergy drug montelukast (Singulair), thereby restricting its use (Food and Drug Administration, 2020; Dahlén et al., 2011). Subsequently, Global Initiative for Asthma (GINA) guidelines explicitly addressed the adverse effects associated with LTRAs in its 2021 report (GINA. Global Initiative for Asthma, 2025).

However, although a substantial amount of clinical research data, particularly from large-scale cohort studies utilizing electronic health records or national databases (Huang et al., 2021; Yao et al., 2025), has suggested a potential association, a previous study (Bai et al., 2023) systematically revealed the risk between leukotriene receptor antagonists and neuropsychiatric entities; however, that study included a relatively small number of studies and did not perform systematic analyses across different age subgroups. Therefore, we conducted a systematic re-evaluation of the relevant studies on the association between LTRAs and neuropsychiatric risks. By synthesizing the available evidence and analyzing the relationship between different age groups and adverse reactions, aiming to provide higher-level evidence for drug safety issues. Such a comprehensive analysis is crucial for guiding clinical decision-making, particularly for vulnerable populations such as children and adolescents.

## Materials and methods

### Registration of review protocol

This study strictly adhered to the 2020 Preferred Reporting Items for Systematic Reviews and Meta-Analyses (PRISMA) guidelines (Page et al., 2021). A systematic review and meta-analysis were conducted to evaluate the association between leukotriene receptor antagonists and neuropsychiatric disorders (Research Checklist_PRISMA). The study protocol was prospectively registered and published on the International Prospective Register of Systematic Reviews (PROSPERO) under registration number CRD420251042206.

### Search strategy

A comprehensive literature search was performed in the PubMed, Web of Science, Embase, Cochrane Library, Pharmacovigilance database and the gray literature for case reports and studies investigating the association between LTRA use and neuropsychiatric disorders. The search encompassed records from the inception of each database until 28 April 2025, with no restrictions on language. The search strategy incorporated terms related to the exposure (e.g., “Leukotriene Antagonists”, “Leukotriene blockers”, “leukotriene receptor antagonist”, “leukotriene modifying agent”, “Montelukast”, “pranlukast”, “zafirlukast”) and the outcome (e.g., “Neuropsychiatric Disorders”, “Neuropsychiatric Events”, “Psychiatric Disorders”, “Mental Disorde”) using both MeSH terms and free-text keywords. Additionally, the reference lists of relevant articles were manually screened to identify any potentially eligible studies missed by the initial electronic search.

Based on the research recommendations, the PECO(S) framework (Morgan et al., 2018) was applied to structure the research question. The Population (P) consisted of patients with respiratory diseases such as asthma and/or allergic rhinitis, with no restrictions on age or sex. The Exposure (E) of interest was the use of LTRAs, including montelukast, zafirlukast, and pranlukast. The Comparator (C) group comprised patients who did not use LTRAs or who used other controller medications, such as inhaled corticosteroids. Regarding the Outcomes (O), the primary outcome was the composite endpoint of overall neuropsychiatric adverse events. Secondary outcomes included various specific neuropsychiatric disorders (e.g., anxiety, depression, sleep disorders and suicidal behavior), which were analyzed with stratification by age. The Study Design (S) was restricted to cohort studies.

**FIGURE 1:**
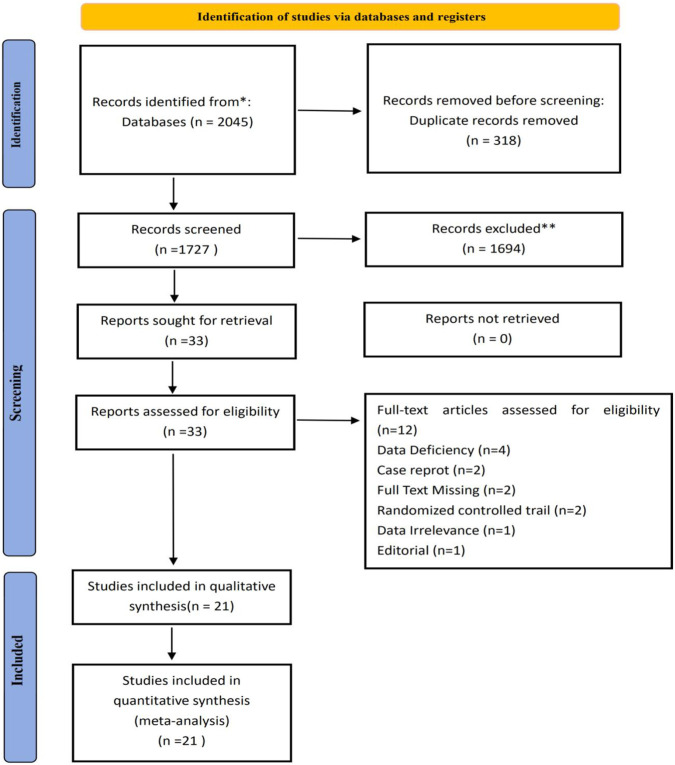
Flow diagram of the literature selection.

The detailed search process and full strategy are provided in Figure 1 and the Supplementary Material.

### Eligibility criteria

#### Inclusion criteria

1. Population: Individuals of any age or gender with underlying respiratory diseases (e.g., asthma, allergic rhinitis) who were prescribed LTRAs.
2. Study design: Observational studies, including cross-sectional, case-control, and cohort designs.
3. Exposure: Use of any LTRA with a clear exposure definition (e.g., at least one prescription dispensed).
4. Outcomes: Studies that reported effect estimates (e.g., relative risk [RR], hazard ratio [HR], odds ratio [OR]) with corresponding 95% confidence intervals (CIs), or provided sufficient raw data (e.g., number of events in exposed and unexposed groups) to allow calculation of these estimates.

#### Exclusion criteria

1. Study design: Randomized controlled trials, reviews, case reports, case series, editorials, commentaries, conference abstracts with incomplete data, and *in vitro* or animal studies.
2. Irrelevant outcomes: Studies that did not report any pre-specified neuropsychiatric outcomes.
3. Unavailable data: Studies from which effect estimates or raw data could not be extracted and were unavailable from the authors upon request.
4. Duplication: Duplicate publications or studies reporting on overlapping cohorts.

### Study selection

The literature screening process was conducted in two stages. Initially, two investigators (JX, SZ) performed a preliminary search in medical databases according to the predefined inclusion and exclusion criteria. All retrieved records were imported into EndNote X9 software, where duplicate studies were removed through both automated and manual processes. The titles and abstracts of the remaining articles were screened against the eligibility criteria to identify potentially relevant studies. In the second stage, full-text articles were thoroughly reviewed to determine their suitability for inclusion in the meta-analysis. Any discrepancies encountered during the screening process were resolved through discussion with a third author (YZ) until consensus was reached.

### Data extraction and quality assessment

Data were extracted from the eligible studies by two investigators (JX, SZ). A narrative synthesis approach was employed for data analysis. The extracted parameters included the first author, publication year, country, study design, data source, drug type, study period, participant age characteristics, disease type, total sample size, outcome definition, and the maximum adjusted effect estimate with its corresponding 95% CI. When multiple publications originated from the same cohort, the following stringent selection strategy was applied: (1) studies were considered independent if they involved distinct population groups based on age; (2) studies were treated separately if they assessed different categories of neuropsychiatric risk within the same cohort.

The methodological quality of the initially included studies was independently assessed by two reviewers (JX, SZ) using the NOS (Chhina, 2024). The NOS instrument comprised eight items across three domains: selection, comparability, and outcome. A maximum of 9 points could be allocated per study, distributed as follows: 4 points for selection, 2 points for comparability, and 3 points for outcome. Based on their total scores, studies were categorized as low (0–3 points), moderate (4–6 points), or high (7–9 points) quality.

Furthermore, the overall certainty of evidence for the included studies was evaluated using the Grading of Recommendations, Assessment, Development, and Evaluations (GRADE) framework. The evidence certainty was rated as “high,” “moderate,” “low,” or “very low.” Any discrepancies in quality assessments were resolved through discussion with a third investigator (YZ) until consensus was reached.

### Data synthesis and analysis

All data analyses and visualizations were performed using R (version 4.2.2). Forest plots were utilized to visually present the RR with 95% confidence intervals from individual studies, along with the pooled overall effect estimates. All included studies employed an observational design and were descriptively summarized according to study type (cohort, case-control, cross-sectional). Due to the low incidence of the outcomes, different effect measures (RRs, HRs, and ORs) were all considered approximate risk ratios and were pooled accordingly in the meta-analysis. When included studies did not report RR measures, the required data were calculated from reported frequency data or obtained by contacting the corresponding authors, with the aim of maximizing the number of studies eligible for meta-analysis.

Given the methodological variations among the included studies, a random-effects model was employed for all analyses. The τ and I^2^ statistics with their 95% confidence intervals served as heterogeneity measures, with I^2^ values of 0%, 25%, 50%, and 75% indicating no, low, moderate, and high heterogeneity, respectively. To further investigate the exposure-outcome relationship, subgroup analyses were conducted based on different age populations. Potential publication bias was assessed through funnel plot visualization and Egger’s regression test. A p-value < 0.05 or visual asymmetry in the funnel plot was considered indicative of potential publication bias, in which case the trim-and-fill method was applied to evaluate its impact on result reliability. Additionally, a leave-one-out sensitivity analysis was performed to assess result robustness and examine the influence of potential confounding factors. This involved systematically excluding studies with high risk of bias to evaluate the contribution of individual studies to the overall effect. The random-effects model results were also compared with fixed-effects model results to ensure the robustness of the findings.

## Results

Figure 1 presents the PRISMA flowchart of the study selection process. A total of 2,045 potentially relevant records were identified through comprehensive database searching and citation screening. After removing 318 duplicates, the titles and abstracts of the remaining records were screened, and full-text articles were retrieved for further assessment. This process led to the exclusion of 1,694 studies that did not meet the predefined inclusion criteria. Subsequently, 33 records were deemed eligible for full-text review following title and abstract assessment. Upon further examination, 12 records were excluded with specific reasons documented in Supplementary Table S1. Consequently, 21 observational studies (Huang et al., 2021; Yao et al., 2025; Fox et al., 2022; Glockler-Lauf et al., 2019; Lei et al., 2025; Tsai et al., 2022; Tsai et al., 2023; Jordan et al., 2023; Haarman et al., 2017)—including cross-sectional, case-control, and cohort designs—were included in the final analysis. The key characteristics of the included studies, including study design and demographic information, are summarized in Table 1.

**TABLE 1:** Characteristics of the studies included in the meta-analysis.

Author, year	Country	Study type	Data from	Drug type	Study period	Age	Disease type	Total	Type of outcome
Christopher W Fox 2022	Canada	Cohort study	The national veterans health administration (VHA) administrative data	Montelukast	2014	—	Asthma	29,292	Overall neuropsychiatric disorders, mood disorders, anxiety, sleep disorders
S dresden Glockler-Lauf 2019	Canada	Case–control study	The Ontario asthma surveillance information system	Montelukast	2004.4.1–2015.3.31	5–18	Asthma	4,395	Overall neuropsychiatric disorders
Po-Yu Huang 2021	China-Taiwan	Cohort study	Taiwan 2010 national health insurance research database	Montelukast	1997.1.1–2013.12.31	1–12	Asthma	54,487	ADHD
Wei-Te Lei 2025	China-Taiwan	Cohort study	The Taiwan national health insurance research database	Montelukast	2004.1.1–2008.12.31	0–18	Asthma	14,588	Overall neuropsychiatric disorders, ADHD, Adjustment Disorders, Affective disorders, anxiety, autism, delay, emotional disorders, personality disorders, psychosis, Schizophrenia, Tourette syndrome, suicide
Min-Lan Tsai 2022	China-Taiwan	Retrospective cohort study	The Taiwan national health insurance research database	Other	2005.1.1–2018.12.31	0–18	Asthma or allergic rhinitis or atopic dermatitis	53,968	Tourette syndrome
Hui-Ju Tsai 2023	China-Taiwan	Cohort study	The Taiwan national health insurance research database	Other	2009–2019	Children	Asthma or allergic rhinitis or atopic dermatitis	576,157	ADHD, autism, Tourette syndrome
Tsung-Chieh Yao 2025	China-Taiwan	Cohort study	The Taiwan national health insurance research database	Other	2010–2021	6–64	Asthma	243,765	Overall neuropsychiatric disorders, psychosis, anxiety, behavioral and emotional disorders, movement disorders, mood disorders, sleep disorders, personality disorders
Alexander Jordan 2023	Danish	Retrospective cohort study	The Danish nationwide health registers	Montelukast	2011.1.1–2018.12.31	>18	Asthma	336,605	Overall neuropsychiatric disorders
Meindina G Haarman 2017	Dutch	Retrospective cohort study	WHO Global database, VigiBase	Montelukast	∼2016.7.13	All	Asthma	17,723	Depression, Aggression, suicide, anxiety, sleep disorders
Young-Woo Jo 2025	Korea	Case–control study	The Korean claims database	Montelukast	2002–2013	>20	Asthma or allergic rhinitis	290,708	Overall neuropsychiatric disorders, suicide
Sang Oh Kang 2021	Korea	Case–control study	The Korean national health insurance Service database	Montelukast	2003–2013	72.7 ± 6.6	Asthma	173,157	Overall neuropsychiatric disorders, sleep disorders, mood disorders, anxiety
Ji Soo Park 2022	Korea	Case–control study	The Korean national health insurance Service	Other	2004.1.1–2018.12.31	3–30	Asthma or allergic rhinitis	17,001	Overall neuropsychiatric disorders, psychosis, mood disorders, anxiety, sleep disorders, Cognitive disorders, movement disorders, personality disorders
Jung-Hyun Kim 2023	Korea	Retrospective population-based study	The Korea’s national health insurance system claims records	Montelukast	2008–2015.6	All	Asthma	93,220	Overall neuropsychiatric disorders, depression, Memory problems, dissociation, Obsessive-compulsive symptoms, attention Problems, sleep disorders, disorientation, anxiety, stress reactions, Somatic symptoms, Hallucinations, Tremors, agitation, restlessness, Irritability, suicide
Jae Won Kim 2024	South Korea	Retrospective cohort study	Health insurance review and assessment (HIRA) dataset	Montelukast	2018.1.1–2021.12.31	0–19	Asthma or allergic rhinitis	806,930	Overall neuropsychiatric disorders
Viktor Wintzell 2025	Sweden	Cohort study	Swedish nation wide data from routine clinical practice	Montelukast	2007.1.1–2021.11.30	6–17	Asthma	74,291	Overall neuropsychiatric disorders, anxiety, depression, sleep disorders, suicide, disrupted control, psychosis
Oznur Yilmaz Bayer 2022	Turkey	Prospective, observational study	Outpatient clinic of the pediatric allergy and asthma department of Gazi University Hospital	Montelukast	2013.9–2014.3	3–18	Asthma	125	Overall neuropsychiatric disorders
Mir M Ali 2015	United States of America	Cohort study	The LifeLink health Plan claims data	Montelukast	1998.1.1- 2009.12.31	0–18	Asthma	7,680	Overall neuropsychiatric disorders
Jingchao Yan 2024	United States of America	Cross-sectional study	The national health and Nutrition examination survey	Other	2007–2016	>20	—	9,414	Depression
Ducharme, F.M 2017	United States of America	Retrospective cohort study	The Sainte-Justine University health Centre	Montelukast	2011.2–2016.4	1–17	Asthma	168	Overall neuropsychiatric disorders
Tapio Paljarvi 2024	United States of America	Cohort study	The TriNetX Analytics network patient repository	Other	2015–2019	3–17	Asthma	107,384	Overall neuropsychiatric disorders, Psychotic, mood disorders, anxiety, sleep disorders, suicide, Externalising symptoms, Internalising symptoms
Tanvi Patil 2023	United States of America	Observational cohort study	The veterans health administration database	Montelukast	2020.1.1–2021.7.1	>18 (with COVID-19)	Asthma	26,249	Depression

Among the observational studies included in this analysis, 16 reported on overall neuropsychiatric disorders (Yao et al., 2025; Fox et al., 2022; Glockler-Lauf et al., 2019; Lei et al., 2025; Jordan et al., 2023; Kim et al., 2024; Paljarvi et al., 2024; Yilmaz Bayer et al., 2022; Ali et al., 2015; Jo et al., 2025; Kang et al., 2021; Kim et al., 2023; Park et al., 2022; Ducharme and Bénard, 2017; Wintzell et al., 2025). The number of studies reporting each specific disorder type was as follows: 9 studies on anxiety (Yao et al., 2025; Fox et al., 2022; Lei et al., 2025; Haarman et al., 2017; Paljarvi et al., 2024; Kang et al., 2021; Kim et al., 2023; Park et al., 2022; Wintzell et al., 2025), 3 on personality disorders (Yao et al., 2025; Lei et al., 2025; Park et al., 2022), 6 on suicide (Lei et al., 2025; Haarman et al., 2017; Paljarvi et al., 2024; Jo et al., 2025; Kim et al., 2023; Wintzell et al., 2025), 5 on psychosis (Yao et al., 2025; Lei et al., 2025; Paljarvi et al., 2024; Park et al., 2022; Wintzell et al., 2025), 5 on mood disorders (Yao et al., 2025; Fox et al., 2022; Paljarvi et al., 2024; Kang et al., 2021; Park et al., 2022), 8 on sleep disorders (Yao et al., 2025; Fox et al., 2022; Haarman et al., 2017; Paljarvi et al., 2024; Kang et al., 2021; Kim et al., 2023; Park et al., 2022; Wintzell et al., 2025), 5 on depression (Haarman et al., 2017; Kim et al., 2023; Wintzell et al., 2025; Patil et al., 2023; Yan et al., 2024), 2 on emotional disorders (Yao et al., 2025; Lei et al., 2025), and 2 on movement disorders (Yao et al., 2025; Park et al., 2022). Attention-deficit/hyperactivity disorder (ADHD) (Huang et al., 2021), Tourette syndrome (Tsai et al., 2022), and autism (Tsai et al., 2023) were exclusively reported in pediatric populations, with one study identified for each of these conditions.

The methodological quality of the included studies was assessed using the NOS. 13 studies were rated as high-quality literature with rigorous methodological design, and 8 studies were considered to be of moderate quality (Supplementary Table S2).

A meta-analysis of 13 primary outcomes was performed. The summarized results of all meta-analyses are presented in Table 2. These included the primary outcome, which was defined as the composite endpoint of overall neuropsychiatric adverse events, and the secondary outcomes, which comprised various specific neuropsychiatric disorders (e.g., anxiety, depression, sleep disorders, and suicidal behavior) and were analyzed with stratification by age. The results demonstrated that LTRA use exhibited distinct neuropsychiatric risk profiles across different age groups. Among adult patients, LTRA administration was associated with several adverse neuropsychiatric events, including anxiety, depression, and sleep disorders. In contrast, no significant increase in comparable risks was observed in the pediatric population, a finding that may be attributed to developmental differences in the blood-brain barrier and drug metabolism.

**TABLE 2:** Summary results of meta-analyses.

Outcome	No. of studies	Pooled RR (95% CI)			I^2^	τ^2^
Overall population						
Overall neuropsychiatric disorders	16	1.11	0.98	1.26	96.90%	0.0322
Anxiety	9	1.25	0.86	1.82	99.70%	0.3195
Personality disorders	3	0.99	0.58	1.69	0%	0
Suicide	6	2.02	0.72	5.66	99.80%	1.4613
Psychosis	5	1.31	0.92	1.87	31.50%	0.0573
Mood disorders	5	1.41	0.92	1.41	96.10%	0.0545
Sleep disorders	8	1.33	0.88	2.01	99.70%	0.3529
Depression	5	1.66	0.78	3.54	99.90%	0.7167
Emotional disorders	2	1.03	0.68	1.57	82.90%	0.0772
Children						
Overall neuropsychiatric disorders	9	1.12	0.9	1.39	91.40%	0.0799
ADHD	1	1.04	0.93	1.17	​	​
Anxiety	3	0.98	0.78	1.22	81.10%	0.0283
Personality disorders	1	1	0.2	4.95	​	​
Tourette syndrome	1	1.36	1.23	1.54	​	​
Suicide	3	1.25	0.99	1.58	0%	0
Psychosis	3	1.03	0.71	1.50	0%	0
Mood disorders	1	1.16	1.05	1.29	​	​
Sleep disorders	2	1.24	0.72	2.15	96.20%	0.1514
Depression	1	1.16	0.7	1.94	​	​
Autism	1	1.01	0.65	1.59	​	​
Adult						
Overall neuropsychiatric disorders	4	1.3	1.08	1.56	96.90%	0.0322
Anxiety	1	1.63	1.50	1.77	​	​
Mood disorders	1	1.65	1.51	1.81	​	​
Sleep disorders	1	1.54	1.42	1.68	​	​
Depression	2	1.2	0.65	2.2	81.60%	0.1594
Undefined population						
Overall neuropsychiatric disorders	3	0.95	0.83	1.08	94.20%	0.0125
Anxiety	5	1.4	0.74	2.64	99.80%	0.5288
Personality disorders	2	0.99	0.57	1.74	0%	0
Suicide	3	3.13	0.46	21.17	99.90%	2.7549
Psychosis	2	1.53	0.98	2.39	46%	0.0481
Mood disorders	3	0.99	0.86	1.14	72.70%	0.0115
Sleep disorders	5	1.33	0.68	2.57	99.80%	0.05702
Depression	2	2.63	0.39	17.63	100%	1.8927
Movement	2	1.2	0.96	1.5	0%	0

### A meta-analysis of overall outcomes

**FIGURE 2:**
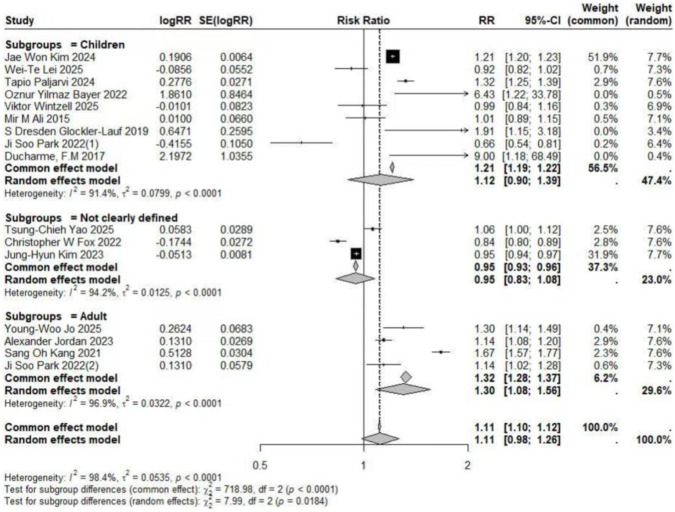
Forest Plot Showing the Association Between Overall Neuropsychiatric Disorders and LTRA. RR: Risk Ratio; CI: Confidence interval. The area of each block is directly proportional to the reciprocal of the variance of the estimated logarithmic hazard ratio (i.e., the weight percentage), and the horizontal lines correspond to the 95% confidence intervals of each study. The vertical axis of the rhombus shows the point estimate of the overall hazard ratio, while the horizontal axis represents its 95% confidence interval. A solid vertical line represents RR = 1.

**FIGURE 3:**
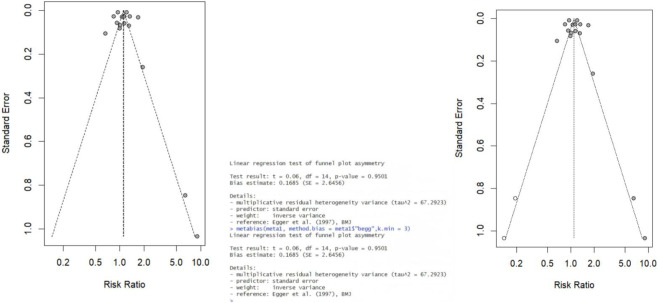
Publication bias and Egger test on Overall Neuropsychiatric Disorders. The funnel plot shows symmetrical distribution of effect estimates, suggesting no evident publication bias (t = 0.06, df = 14, p = 0.9501). Egger’s test confirms this, with a non-significant p-value (p = 0.9501), supporting the robustness of the meta-analysis findings.

**FIGURE 4:**
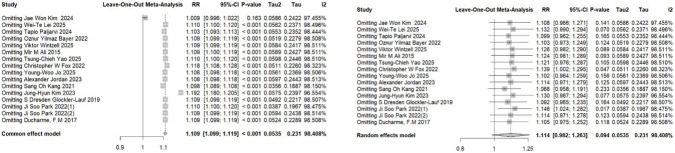
Sensitivity analysis between Overall Neuropsychiatric Disorders and LTRA. Left: Common effect model; Right: Random effects model. RR: Risk Ratio; CI: Confidence interval. The pooled risk estimate remained consistent in direction and magnitude across most leave-one-out analyses. Notably, the exclusion of Jung-Hyun Kim 2023 under the common effect model substantially increased the point estimate (RR = 1.192), while its exclusion under the random effects model did not alter the overall interpretation. The omission of Christopher W Fox 2022 and Ji Soo Park 2022 (1) under the random effects model strengthened the statistical significance (p = 0.047 and p = 0.017, respectively), suggesting these studies contributed to the uncertainty in the primary analysis. The overall findings support the robustness of the meta-analysis conclusion.

The pooled analysis of studies examining the association between LTRA use and neuropsychiatric risk in patients with allergic asthma/rhinitis encompassed 59 estimates from 18 publications (Yao et al., 2025; Fox et al., 2022; Glockler-Lauf et al., 2019; Lei et al., 2025; Jordan et al., 2023; Haarman et al., 2017; Kim et al., 2024; Paljarvi et al., 2024; Yilmaz Bayer et al., 2022; Ali et al., 2015; Jo et al., 2025; Kang et al., 2021; Kim et al., 2023; Park et al., 2022; Ducharme and Bénard, 2017; Wintzell et al., 2025; Patil et al., 2023; Yan et al., 2024). The forest plot revealed a non-significant trend toward increased risk for overall neuropsychiatric disorders (RR = 1.11, 95% CI: 0.98–1.26). Substantial heterogeneity was observed across studies (τ^2^ = 0.0322, I^2^ = 96.9%) (Figure 2). Assessment of funnel plot symmetry using Egger’s linear regression test (Figure 3) revealed no significant publication bias (t = 0.06, df = 14, p = 0.9501). This finding was consistent with Egger’s test results (p = 0.9501), indicating a low probability of substantial publication bias affecting the overall findings. Sensitivity analysis (Figure 4) demonstrated that under the random-effects model, the pooled RR range remained between 1.068 and 1.146 when individual studies were sequentially excluded. The direction of association remained largely unchanged in most scenarios, suggesting relative robustness of the results. Notably, exclusion of Ji Soo Park 2022 (1) resulted in a statistically significant association (RR = 1.146, 95% CI: 1.024–1.282, p = 0.017), potentially attributable to its limited effective sample size and deviation from the overall risk trend.

**FIGURE 5:**
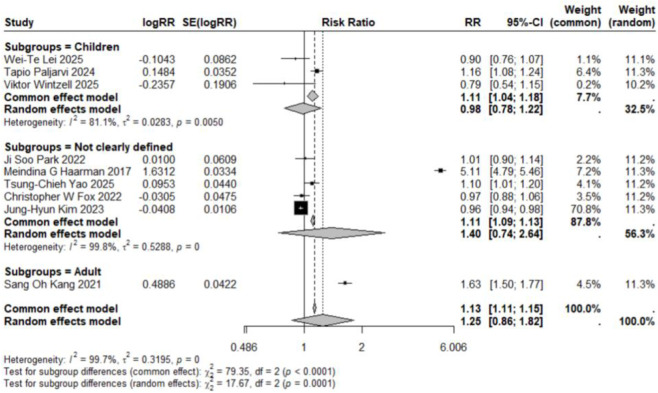
Forest Plot Showing the Association Between Anxiety and LTRA. RR: Risk Ratio; CI: Confidence interval. The area of each block is directly proportional to the reciprocal of the variance of the estimated logarithmic hazard ratio (i.e., the weight percentage), and the horizontal lines correspond to the 95% confidence intervals of each study. The vertical axis of the rhombus shows the point estimate of the overall hazard ratio, while the horizontal axis represents its 95% confidence interval. A solid vertical line represents RR = 1.

**FIGURE 6:**
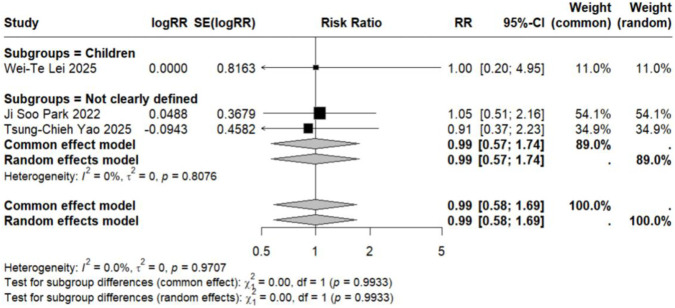
Forest Plot Showing the Association Between Personality Disorders and LTRA. RR: Risk Ratio; CI: Confidence interval. The area of each block is directly proportional to the reciprocal of the variance of the estimated logarithmic hazard ratio (i.e., the weight percentage), and the horizontal lines correspond to the 95% confidence intervals of each study. The vertical axis of the rhombus shows the point estimate of the overall hazard ratio, while the horizontal axis represents its 95% confidence interval. A solid vertical line represents RR = 1.

**FIGURE 7:**
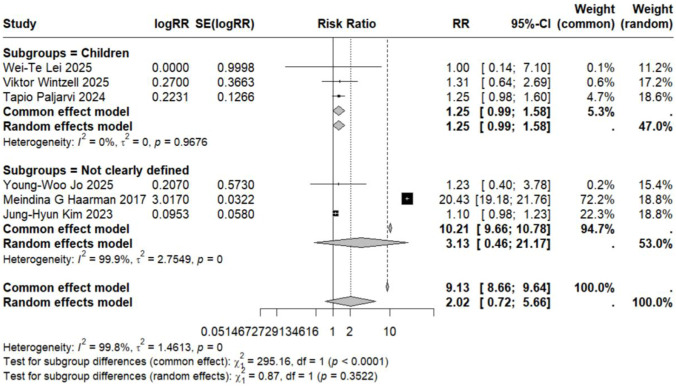
Forest Plot Showing the Association Between Suicide and LTRA. RR: Risk Ratio; CI: Confidence interval. The area of each block is directly proportional to the reciprocal of the variance of the estimated logarithmic hazard ratio (i.e., the weight percentage), and the horizontal lines correspond to the 95% confidence intervals of each study. The vertical axis of the rhombus shows the point estimate of the overall hazard ratio, while the horizontal axis represents its 95% confidence interval. A solid vertical line represents RR = 1.

**FIGURE 8:**
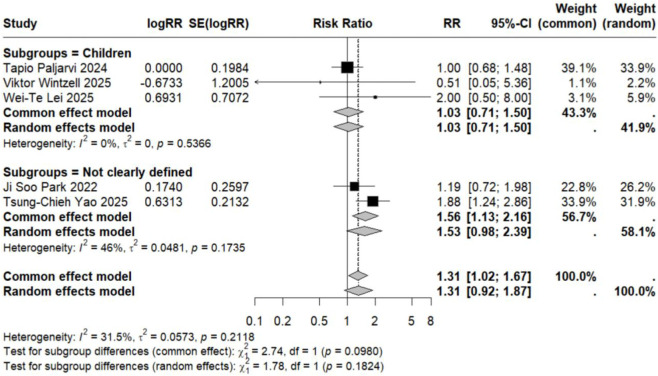
Forest Plot Showing the Association Between Psychosis and LTRA. RR: Risk Ratio; CI: Confidence interval. The area of each block is directly proportional to the reciprocal of the variance of the estimated logarithmic hazard ratio (i.e., the weight percentage), and the horizontal lines correspond to the 95% confidence intervals of each study. The vertical axis of the rhombus shows the point estimate of the overall hazard ratio, while the horizontal axis represents its 95% confidence interval. A solid vertical line represents RR = 1.

**FIGURE 9:**
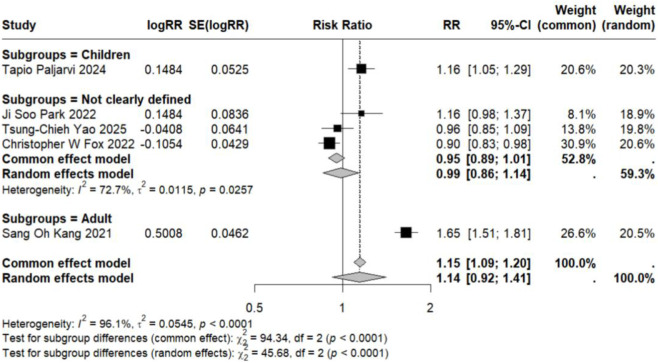
Forest Plot Showing the Association Between Mood Disorders and LTRA. RR: Risk Ratio; CI: Confidence interval. The area of each block is directly proportional to the reciprocal of the variance of the estimated logarithmic hazard ratio (i.e., the weight percentage), and the horizontal lines correspond to the 95% confidence intervals of each study. The vertical axis of the rhombus shows the point estimate of the overall hazard ratio, while the horizontal axis represents its 95% confidence interval. A solid vertical line represents RR = 1.

**FIGURE 10:**
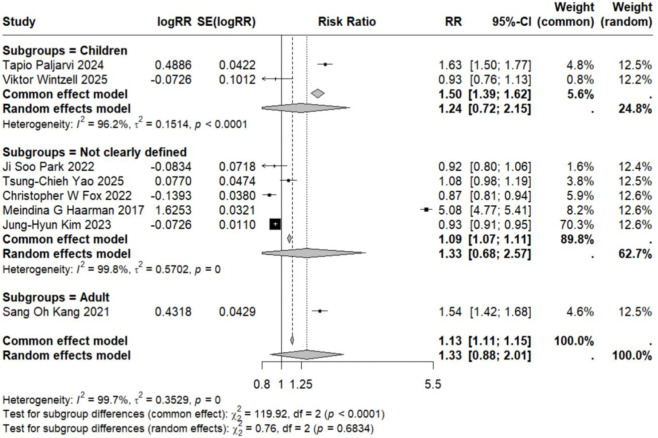
Forest Plot Showing the Association Between Sleep Disorders and LTRA. RR: Risk Ratio; CI: Confidence interval. The area of each block is directly proportional to the reciprocal of the variance of the estimated logarithmic hazard ratio (i.e., the weight percentage), and the horizontal lines correspond to the 95% confidence intervals of each study. The vertical axis of the rhombus shows the point estimate of the overall hazard ratio, while the horizontal axis represents its 95% confidence interval. A solid vertical line represents RR = 1.

**FIGURE 11:**
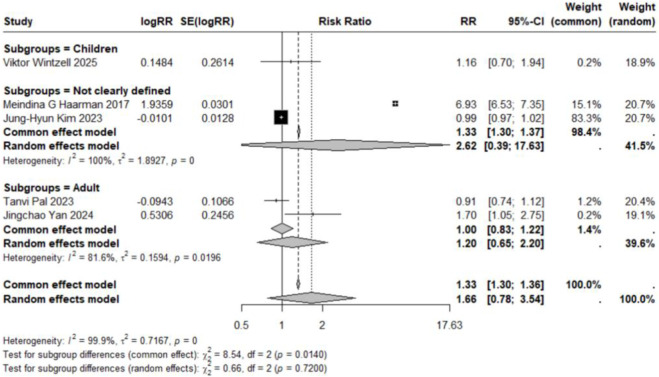
Forest Plot Showing the Association Between Depression and LTRA. RR: Risk Ratio; CI: Confidence interval. The area of each block is directly proportional to the reciprocal of the variance of the estimated logarithmic hazard ratio (i.e., the weight percentage), and the horizontal lines correspond to the 95% confidence intervals of each study. The vertical axis of the rhombus shows the point estimate of the overall hazard ratio, while the horizontal axis represents its 95% confidence interval. A solid vertical line represents RR = 1.

**FIGURE 12:**
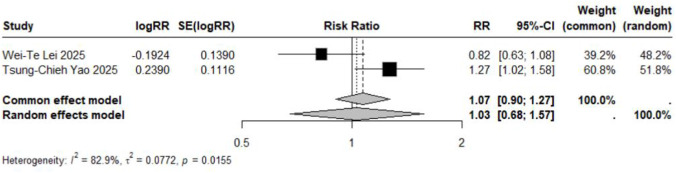
Forest Plot Showing the Association Between Emotional Disorders and LTRA. RR: Risk Ratio; CI: Confidence interval. The area of each block is directly proportional to the reciprocal of the variance of the estimated logarithmic hazard ratio (i.e., the weight percentage), and the horizontal lines correspond to the 95% confidence intervals of each study. The vertical axis of the rhombus shows the point estimate of the overall hazard ratio, while the horizontal axis represents its 95% confidence interval. A solid vertical line represents RR = 1.

Subgroup analyses based on specific neuropsychiatric disorder types yielded pooled RR estimates ranging from 0.89 to 2.02. No statistically significant associations were observed for anxiety, personality disorders, suicide, psychosis, mood disorders, sleep disorders, depression, or emotional disorders. Although these results did not reach statistical significance, all point estimates exceeded zero (RR > 0), suggesting potential elevated risks that warrant consideration (Figures 5–12).

### Association in the pediatric population

The pooled analysis of studies investigating the association between LTRA use and neuropsychiatric risk in the pediatric population encompassed 26 estimates from 12 publications (Huang et al., 2021; Glockler-Lauf et al., 2019; Lei et al., 2025; Tsai et al., 2022; Tsai et al., 2023; Kim et al., 2024; Paljarvi et al., 2024; Yilmaz Bayer et al., 2022; Ali et al., 2015; Park et al., 2022; Ducharme and Bénard, 2017; Wintzell et al., 2025). No statistically significant association was observed between LTRA use and overall neuropsychiatric disorders in children (RR = 1.12, 95% CI: 0.90–1.39; I^2^ = 91.4%, τ^2^ = 0.0799).

Analysis of specific neuropsychiatric disorders revealed no significant associations for most conditions, including ADHD (n = 1) (Huang et al., 2021), anxiety (n = 3) (Lei et al., 2025; Paljarvi et al., 2024; Wintzell et al., 2025), suicide (n = 3) (Lei et al., 2025; Paljarvi et al., 2024; Wintzell et al., 2025), sleep disorders (n = 2) (Paljarvi et al., 2024; Wintzell et al., 2025), personality disorders (n = 1) (Lei et al., 2025), depression (n = 1) (Wintzell et al., 2025), or autism (n = 1) (Tsai et al., 2023) (detailed in Table 2; Figures 5–7, 10, 11). One study reported a significant association between LTRA use and mood disorders (RR = 1.16, 95% CI: 1.05–1.29) (Paljarvi et al., 2024). Another study indicated that LTRA use (particularly montelukast) in children with asthma, allergic rhinitis, or atopic dermatitis was associated with an increased risk of Tourette syndrome (RR = 1.36, 95% CI: 1.23–1.54) (Tsai et al., 2022). However, overall analysis revealed no clear evidence of a significant association between LTRA exposure and neuropsychiatric risk in the pediatric population.

### Association in the adult population

In contrast to the pediatric population, the use of LTRAs in adults was associated with a statistically significant increase in neuropsychiatric risk. The pooled analysis of 9 estimates from 6 publications (Jordan et al., 2023; Jo et al., 2025; Kang et al., 2021; Park et al., 2022; Patil et al., 2023; Yan et al., 2024) revealed an overall risk ratio of 1.30 (95% CI: 1.08–1.56), with considerable heterogeneity observed across studies (I^2^ = 96.9%, τ^2^ = 0.0322). Analysis of specific disorders revealed significant positive associations for most conditions. One study (Kang et al., 2021) conducted in elderly asthma patients found that montelukast use was associated with an increased risk of mood disorders (RR = 1.65, 95% CI: 1.51–1.81). The same study also identified elevated risks for sleep disorders (RR = 1.54, 95% CI: 1.42–1.68) and anxiety (RR = 1.63, 95% CI: 1.50–1.77). In contrast, two studies (Patil et al., 2023; Yan et al., 2024) reporting on depression-related outcomes showed no statistically significant association with LTRA use (RR = 1.20, 95% CI: 0.65–2.20) (Figures 3, 5, 9–11).

### Association in studies with undefined age populations

**FIGURE 13:**
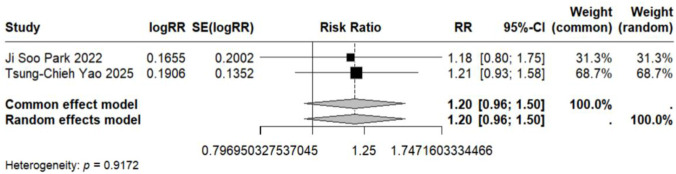
Forest Plot Showing the Association Between Movement and LTRA. RR: Risk Ratio; CI: Confidence interval. The area of each block is directly proportional to the reciprocal of the variance of the estimated logarithmic hazard ratio (i.e., the weight percentage), and the horizontal lines correspond to the 95% confidence intervals of each study. The vertical axis of the rhombus shows the point estimate of the overall hazard ratio, while the horizontal axis represents its 95% confidence interval. A solid vertical line represents RR = 1.

The pooled analysis of 27 estimates from 6 studies (Yao et al., 2025; Fox et al., 2022; Haarman et al., 2017; Jo et al., 2025; Kim et al., 2023; Park et al., 2022) involving populations with undefined age ranges demonstrated no significant overall association (RR = 0.95, 95% CI: 0.83–1.08; τ^2^ = 0.0125, I^2^ = 94.2%). For several severe outcomes, including suicide (n = 3) (RR = 3.13, 95% CI: 0.46–21.17; τ^2^ = 2.7549, I^2^ = 99.9%) (Haarman et al., 2017; Jo et al., 2025; Kim et al., 2023) and depression (n = 2) (RR = 2.63, 95% CI: 0.39–17.63; τ^2^ = 1.8927, I^2^ = 100%) (Haarman et al., 2017; Kim et al., 2023), substantial point estimates with wide confidence intervals were observed, indicating considerable uncertainty and heterogeneity; none of these associations reached statistical significance. The remaining outcomes also showed no statistically significant associations, as detailed in Figures 2, 5–11, 13. Future studies are warranted to substantiate these findings.

### Sensitivity analysis

Sensitivity analyses were performed for the primary outcomes, including anxiety, suicide, mood disorders, sleep disorders, depression, and psychosis (Supplementary Figures S1-S6). The results for anxiety remained robust across the sensitivity analysis. In contrast, the pooled estimates for depression, mood disorders, sleep disorders, suicide, and psychosis were highly sensitive to the sequential exclusion of individual studies under the random-effects model. The overall non-significant findings for these outcomes were unstable and substantially influenced by specific studies.

### Publication bias

Publication bias was assessed for the primary outcomes, including anxiety, suicide, mood disorders, sleep disorders, depression, and psychosis (Supplementary Figures S7-S12). For psychosis, the funnel plot demonstrated approximately symmetric distribution of effect estimates around the pooled mean, with Egger’s test showing no significant publication bias (P = 0.9070 > 0.05). The funnel plot for anxiety exhibited mild asymmetry, while those for depression, mood disorders, sleep disorders, and suicide revealed substantial asymmetry. Additional trim-and-fill analyses were performed, with all Egger’s tests yielding p-values greater than 0.05. This pattern suggested that the observed asymmetry was more likely attributable to substantial heterogeneity clinical diversity among the limited number of studies, studies of low methodological quality, and genuine differences in effect sizes, rather than to publication bias. Due to the limited number of studies available for other neuropsychiatric outcomes, neither publication bias nor sensitivity analyses were conducted for those endpoints.

### Evidence appraisal using GRADE

The quality of evidence for each health outcome was evaluated and graded as “high,” “moderate,” “low,” or “very low” according to established criteria. Based on these evidence ratings and classification standards, the study findings were systematically summarized, with detailed results presented in Supplementary Table S3. Compared to randomized controlled trials, observational studies were typically assigned more stringent GRADE ratings due to inherent design limitations. The majority of outcomes in this analysis received a Grade C evidence level, which adequately supports the observed association between LTRA use and neuropsychiatric risk.

## Discussion

### Summary of evidence

This study comprehensively evaluated the association between LTRAs and neuropsychiatric disorders. It represented the first systematic analysis to perform age-stratified subgroup assessments, thereby providing higher-level evidence and crucial data for age-specific safety considerations in LTRA therapy. Through pooled evaluation of 21 included studies and risk analysis of 12 primary outcomes, the neuropsychiatric safety profile of LTRAs was thoroughly examined. The findings regarding age-specific risk differences were particularly noteworthy, revealing distinct risk-benefit profiles between pediatric and adult populations that carried significant implications for clinical practice and pharmacovigilance monitoring.

In this systematic review and meta-analysis, the overall trend of the pooled results demonstrated differential associations across subgroups. The use of LTRAs in adult patients was associated with adverse neuropsychiatric events including anxiety, mood disorders and sleep disorders. In contrast, no statistically significant increase in comparable risks was observed in the pediatric population.

According to the GINA guidelines, while LTRAs were not recommended as first-line therapy for adolescents and adults aged 12 years and older, they remained alternative controller options at Step 2 and Step 3 for children under 11 years, in addition to serving as alternative add-on therapy at Step 4 (Bateman et al., 2008). Our study provided evidence-based safety data and further supporting evidence for clinical guideline implementation. The absence of statistically significant associations between LTRA use and most neuropsychiatric outcomes—including ADHD, anxiety, suicide, and sleep disorders—indicated a favorable safety profile in the pediatric population. However, it is noteworthy that our analysis identified increased risks for Tourette syndrome (RR = 1.36, 95% CI: 1.23–1.54) and mood disorders (RR = 1.16, 95% CI: 1.05–1.29). This specific pattern suggested potential underlying biological mechanisms. The elevated tic risk might originate from the impact of leukotriene pathways on basal ganglia circuitry, where LTRAs could disrupt dopaminergic and other neurotransmitter balances, potentially leading to abnormal motor control (Baldermann et al., 2025; Leckman et al., 2006). The mood disorder risk may be related to LTRA-mediated modulation of central neuroinflammation (Sharma et al., 2024). The absence of significantly increased risks for other neuropsychiatric outcomes indicated disease-specific effects of LTRAs, suggesting that the affected neuroimmune and neurotransmitter pathways are more closely associated with the core circuits regulating tics and mood, rather than broadly influencing the pathological basis of all psychiatric disorders (Shabab et al., 2017).

In the adult population, we observed markedly different outcomes compared to the pediatric group, with LTRA use demonstrating an increased risk of neuropsychiatric disorders. Administration of LTRAs was associated with elevated incidence of overall neuropsychiatric adverse events (RR = 1.30, 95% CI: 1.08–1.56), primarily manifested as significantly increased risks of mood disorders (RR = 1.65, 95% CI: 1.51–1.81), sleep disorders (RR = 1.54, 95% CI: 1.42–1.68) and anxiety (RR = 1.63, 95% CI: 1.50–1.77). These findings suggested that the biological mechanisms underlying LTRA-associated neuropsychiatric effects were potentially age-dependent, possibly involving developmental differences in blood-brain barrier permeability, drug metabolism, and central nervous system receptor expression patterns across different age groups (Marques et al., 2022). These developmental factors collectively influenced the distribution characteristics, steady-state concentrations, and pharmacological effects of LTRAs on the central nervous system in pediatric versus adult populations, thereby partially explaining the age-specific patterns of neuropsychiatric risk signals.

At the mechanistic level, we hypothesized that LTRAs might participate in neuropsychiatric pathophysiological processes through direct or indirect pathways, including modulation of central neuroinflammatory responses, alteration of hypothalamic-pituitary-adrenal axis function, and disruption of neurotransmitter system balance (Hunt et al., 2015), thereby particularly contributing to the development of mood and sleep disorders (Felger and Lotrich, 2013; Umetsu et al., 2021). For neuropsychiatric outcomes that did not demonstrate significant associations, such as ADHD, anxiety, and suicide, their manifestation appeared to be determined by multiple factors beyond purely pharmacological effects. Individual psychological status, social support systems, stress levels, and other acquired behavioral and environmental factors collectively constituted the “background risk” for disease manifestation, which may have attenuated or masked subtle drug-specific effects on certain conditions. However, as this study employed an observational design, the findings indicated associations only and could not be used to infer causality. The observed age-specific differences require further validation through basic research and studies with prospective designs.

### Advantages and limitations

This study comprehensively evaluated the association between LTRAs and neuropsychiatric disorders. It represented the first systematic analysis to perform age-stratified subgroup assessments, thereby providing updated evidence-based insights into drug safety profiles. By integrating all relevant studies while rigorously adhering to methodological standards, the analysis employed robust statistical models, extensive sensitivity analyses, and comprehensive publication bias assessments, which collectively ensured the reliability of the findings and clearly established the association between LTRAs and neuropsychiatric risk. Furthermore, the focus on age-stratified analysis ultimately demonstrated the critical necessity of subgroup analyses based on age differentiation.

However, several limitations warrant consideration in this study. The primary limitations stemmed from the inherent constraints of observational study designs. Although the original studies adjusted for various confounders, residual confounding—potentially arising from underlying disease severity, concomitant medications, or genetic factors—remained a plausible alternative explanation for the observed associations, particularly in the adult analyses. To mitigate this concern, we excluded studies that failed to adjust for key confounding variables and conducted multiple sensitivity analyses to evaluate the potential impact of residual confounding. Second, considerable statistical heterogeneity was observed across studies (I^2^ > 90% for many outcomes), which could be attributed to several factors. Most included studies utilized health insurance claims databases, which lacked information for adjusting variables such as underlying disease status and genetic background, potentially introducing bias into the results. To address this, we performed subgroup and sensitivity analyses to minimize the influence of such bias. Third, funnel plot asymmetry observed for certain outcomes suggested the possibility of publication bias, which might have affected the pooled effect estimates. This could be explained by methodological variations across studies, including differences in follow-up duration, participant characteristics, underlying diseases, and age distributions. We therefore employed multiple approaches to assess publication bias, including visual inspection of funnel plots and Egger’s regression test (p < 0.05). Additionally, the trim-and-fill method was applied to adjust for potential publication bias, confirming the stability of the meta-analysis results. Fourth, the comparator framework of this study possessed inherent heterogeneity, a common limitation in observational pharmacoepidemiological studies based on real-world data. This diversity in comparators was also present in previously published meta-analyses of a similar nature (Jobanputra et al., 2023; Jiang et al., 2024; Alves et al., 2012), reflecting the reality of inherent heterogeneity in real-world clinical practice. Fifth, geographic stratification was not performed in this study. Although the included studies were derived from multiple regions, including Asia, Europe, and North America, the number of studies per region was limited, and region was confounded with age subgroups, making it difficult to isolate the independent effects of these two factors. Future studies with more accumulated evidence across different regions may further explore the potential influence of geographic factors on LTRA-related neuropsychiatric risks through meta-regression or network meta-analysis. Finally, the limited number of studies available for certain specific outcomes (such as autism and mood disorders) constrained the precision and certainty of the pooled results. Future research should focus on neuropsychiatric symptoms potentially associated with long-term LTRA use, including agitation, mood disorders, and sleep disturbances. Despite these limitations, the main constraints originated from the design of the primary studies rather than the methodological approaches employed in our analysis. Importantly, our findings provided valuable evidence regarding the association between LTRAs and neuropsychiatric risks.

## Conclusion

Our meta-analysis, which incorporated high-quality observational studies with substantial sample sizes, revealed significant age-dependent differences in the association between LTRA use and neuropsychiatric risk. The comprehensive analysis indicated that LTRA administration in the pediatric population demonstrated a favorable safety profile, showing no significant association with the vast majority of neuropsychiatric disorders. These findings supported its position as a first-line treatment for asthma and allergic rhinitis in children. In contrast, LTRA use in adult patients was significantly associated with increased neuropsychiatric risk. Consequently, the findings suggested that differentiated risk management strategies were warranted in clinical practice: LTRA could be confidently prescribed with ongoing monitoring in pediatric patients, while adult patients required cautious prescription practices, including clear communication of potential risks and maintained vigilance during treatment. This study provided crucial evidence to inform age-specific safety considerations in LTRA pharmacotherapy.

## Data Availability

The original contributions presented in the study are included in the article/Supplementary Material, further inquiries can be directed to the corresponding author.
