# Supplementary material for: Association between leukotriene receptor antagonists and neuropsychiatric disorders: a systematic review and meta-analysis
Source: Front Pharmacol. 2026 Apr 30;17:1787744. doi: 10.3389/fphar.2026.1787744 (PMC13171479; doi:10.3389/fphar.2026.1787744)
Supplement: Supplementary file 1 [file Supplementaryfile1.docx]

Supplementary catalogue

[Search strategies 1](#_Toc23814)

[eFigure 1: Sensitivity analysis between Anxiety and LTRA. 4](#_Toc30963)

[eFigure 2: Sensitivity analysis between Suicide and LTRA. 5](#_Toc372)

[eFigure 3: Sensitivity analysis between Psychosis and LTRA. 6](#_Toc2055)

[eFigure 4: Sensitivity analysis between Mood Disorders and LTRA. 7](#_Toc13626)

[eFigure 5: Sensitivity analysis between Sleep Disorders and LTRA. 8](#_Toc18253)

[eFigure 6: Sensitivity analysis between Depression and LTRA. 9](#_Toc28355)

[eFigure 7: Publication bias and Egger test on Anxiety. 10](#_Toc8001)

[eFigure 8: Publication bias and Egger test on Suicide. 11](#_Toc6481)

[eFigure 9: Publication bias and Egger test on Psychosis. 12](#_Toc18422)

[eFigure 10: Publication bias and Egger test on Mood Disorders. 13](#_Toc26426)

[eFigure 11: Publication bias and Egger test on Sleep Disorders. 14](#_Toc17097)

[eFigure 12: Publication bias and Egger test on Depression. 15](#_Toc4925)

[eTable 1. List of excluded references and reasons for exclusion. 16](#_Toc2795)

[eTable 2. NEWCASTLE - OTTAWA QUALITY ASSESSMENT SCALE (COHORT STUDIES) 17](#_Toc29041)

[eTable 3. Grade Grading Details 19](#_Toc31517)

# Search strategies

| **Database** | **Step** | **Terms** | **Results** |
| --- | --- | --- | --- |
| **PubMed** | 1 | （"Leukotriene Antagonists"[MeSH Terms] OR ("antagonists leukotriene"[Title/Abstract] OR "leukotriene antagonist"[Title/Abstract] OR "antagonist leukotriene"[Title/Abstract] OR "leukotriene receptor antagonist"[Title/Abstract] OR (("Antagonist"[All Fields] OR "antagonists and inhibitors"[MeSH Subheading] OR ("Antagonists"[All Fields] AND "inhibitors"[All Fields]) OR "antagonists and inhibitors"[All Fields] OR "Antagonists"[All Fields]) AND "leukotriene receptor"[Title/Abstract]) OR "receptor antagonist leukotriene"[Title/Abstract] OR "leukotriene receptor antagonists"[Title/Abstract] OR "antagonists leukotriene receptor"[Title/Abstract] OR "receptor antagonists leukotriene"[Title/Abstract]) | 4700 |
|  | 2 | （"Mental Disorders"[MeSH Terms] OR ("neuropsychiatric disorders"[Title/Abstract] OR "neuropsychiatric events"[Title/Abstract] OR "mental disorder"[Title/Abstract] OR "psychiatric disorders"[Title/Abstract] OR "psychiatric disorder"[Title/Abstract] OR "psychiatric diseases"[Title/Abstract] OR "psychiatric disease"[Title/Abstract] OR "psychiatric illness"[Title/Abstract] OR "psychiatric illnesses"[Title/Abstract] OR "mental illness"[Title/Abstract] OR "illness mental"[Title/Abstract] OR "mental illnesses"[Title/Abstract] OR "behavior disorders"[Title/Abstract] OR "diagnosis psychiatric"[Title/Abstract] OR "psychiatric diagnosis"[Title/Abstract] OR "mental disorders severe"[Title/Abstract] OR "mental disorder severe"[Title/Abstract] OR "severe mental disorder"[Title/Abstract] OR ((("sever"[All Fields] OR "Severe"[All Fields] OR "severed"[All Fields] OR "severely"[All Fields] OR "severer"[All Fields] OR "severes"[All Fields] OR "severing"[All Fields] OR "severities"[All Fields] OR "severity"[All Fields] OR "severs"[All Fields]) AND ("Mental"[All Fields] OR "mentalities"[All Fields] OR "mentality"[All Fields] OR "mentalization"[MeSH Terms] OR "mentalization"[All Fields] OR "mentalizing"[All Fields] OR "mentalize"[All Fields] OR "mentalized"[All Fields] OR "mentally"[All Fields])) | 1632474 |
|  | 3 | #1 AND #2 | **73** |
| **Embase** | 1 | leukotriene receptor blocking agent'/exp OR 'leucotriene antagonists/inhibitors':ti,ab,kw OR 'leukotriene antagonist':ti,ab,kw OR 'leukotriene antagonists':ti,ab,kw OR 'leukotriene blocker':ti,ab,kw OR 'leukotriene blocking agent':ti,ab,kw OR 'leukotriene inhibitor':ti,ab,kw OR 'leukotriene receptor antagonist':ti,ab,kw OR 'leukotriene receptor blocker':ti,ab,kw OR 'leukotriene receptor blocking agent':ti,ab,kw | 26879 |
|  | 2 | mental disease'/exp OR 'abnormal mental state':ti,ab,kw OR 'disease, mental':ti,ab,kw OR 'diseased mental state':ti,ab,kw OR 'disorder, mental':ti,ab,kw OR 'disordered mental state':ti,ab,kw OR 'disturbed mental state':ti,ab,kw OR 'illness, mental':ti,ab,kw OR 'insanity':ti,ab,kw OR 'mental abnormality':ti,ab,kw OR 'mental change':ti,ab,kw OR 'mental confusion':ti,ab,kw OR 'mental defect':ti,ab,kw OR 'mental disorder':ti,ab,kw OR 'mental disorders':ti,ab,kw OR 'mental disorders diagnosed in childhood':ti,ab,kw OR 'mental disturbance':ti,ab,kw OR 'mental health condition':ti,ab,kw OR 'mental health disease':ti,ab,kw OR 'mental health disorder':ti,ab,kw OR 'mental health issue':ti,ab,kw OR 'mental health problem':ti,ab,kw OR 'mental illness':ti,ab,kw OR 'mental insufficiency':ti,ab,kw OR 'mental symptom':ti,ab,kw OR 'mentally ill':ti,ab,kw OR 'neurodevelopmental disorder':ti,ab,kw OR 'neurodevelopmental disorders':ti,ab,kw OR 'neuropsychiatric disease':ti,ab,kw OR 'neuropsychiatric diseases':ti,ab,kw OR 'neuropsychiatric disorder':ti,ab,kw OR 'neuropsychiatric disorders':ti,ab,kw OR 'psychiatric disease':ti,ab,kw OR 'psychiatric disorder':ti,ab,kw OR 'psychiatric illness':ti,ab,kw OR 'psychiatric symptom':ti,ab,kw OR 'psychic disease':ti,ab,kw OR 'psychic disorder':ti,ab,kw OR 'psychic disturbance':ti,ab,kw OR 'psychologic disorder':ti,ab,kw OR 'psychologic disturbance':ti,ab,kw OR 'psychological disorder':ti,ab,kw OR 'psychological disturbance':ti,ab,kw OR 'psychopathology':ti,ab,kw OR 'mental disease':ti,ab,kw | 3399985 |
|  | 3 | #1 AND #2 | **1886** |
| **Web of Science** | 1 | TS=（‘Leukotriene Antagonists’ OR ‘antagonists leukotriene’ OR ‘leukotriene antagonist’ OR ‘antagonist leukotriene’ OR ‘leukotriene receptor antagonist’ OR ‘Antagonist'[All Fields] OR 'antagonists and inhibitors’ OR ‘Antagonists’ AND ‘inhibitors’ OR ‘antagonists and inhibitors’ OR ‘Antagonists’ AND ‘leukotriene receptor’ OR ‘receptor antagonist leukotriene’ OR ‘'leukotriene receptor antagonists’ OR ‘antagonists leukotriene receptor’ OR ‘receptor antagonists leukotrienes’) | 1802 |
|  | 2 | TS=（‘Mental Disorders’ OR ‘Neuropsychiatric Disorders’ OR ‘Neuropsychiatric Events’ OR ‘Mental Disorder’ OR ‘Psychiatric Disorders’ OR ‘Psychiatric Disorder’ OR ‘Psychiatric Diseases’ OR ‘Psychiatric Disease’ OR ‘Psychiatric Illness’ OR ‘Psychiatric Illnesses’ OR ‘Mental Illness’ OR ‘Illness, Mental’ OR ‘Mental Illnesses’ OR ‘Behavior Disorders’ OR ‘Diagnosis, Psychiatric’ OR ‘Psychiatric Diagnosis’ OR ‘Mental Disorders, Severe’ OR ‘Mental Disorder, Severe’ OR ‘Severe Mental Disorder’ OR ‘Severe Mental Disorders’) | 379274 |
|  | 3 | #1 AND #2 | **74** |
| **Cochrane Library** | 1 | MeSH descriptor: [Leukotriene Antagonists] explode all trees OR MeSH descriptor: [Antagonist, Leukotriene] explode all trees OR (‘Receptor Antagonist, Leukotriene’ OR ‘Leukotriene Antagonist’ OR ‘Antagonists, Leukotriene Receptor’ OR ‘Antagonist, Leukotriene Receptor’ OR ‘Leukotriene Receptor Antagonist’ OR ‘Antagonists, Leukotriene’ OR ‘Leukotriene Receptor Antagonists’ OR ‘Receptor Antagonists, Leukotriene’):ti,ab,kw | 1222 |
|  | 2 | MeSH descriptor: [Mental Disorders] explode all trees OR MeSH descriptor: [Behavior Disorders] explode all trees OR (‘Mental Disorders, Severe’ OR ‘Severe Mental Disorders’ OR ‘Mental Disorder, Severe’ OR ‘Severe Mental Disorder’ OR ‘Psychiatric Disease’ OR ‘Mental Disorder’ OR ‘Psychiatric Disorders’ OR ‘Psychiatric Illnesses’ OR ‘Psychiatric Diseases’ OR ‘Psychiatric Illness’ OR ‘Mental Illness’ OR ‘Mental Illnesses’ OR ‘Psychiatric Disorder’ OR ‘Illness, Mental’ OR ‘Psychiatric Diagnosis’ OR ‘Diagnosis, Psychiatric’):ti,ab,kw | 160548 |
|  | 3 | #1 AND #2 | **12** |

eFigure 1: Sensitivity analysis between Anxiety and LTRA.


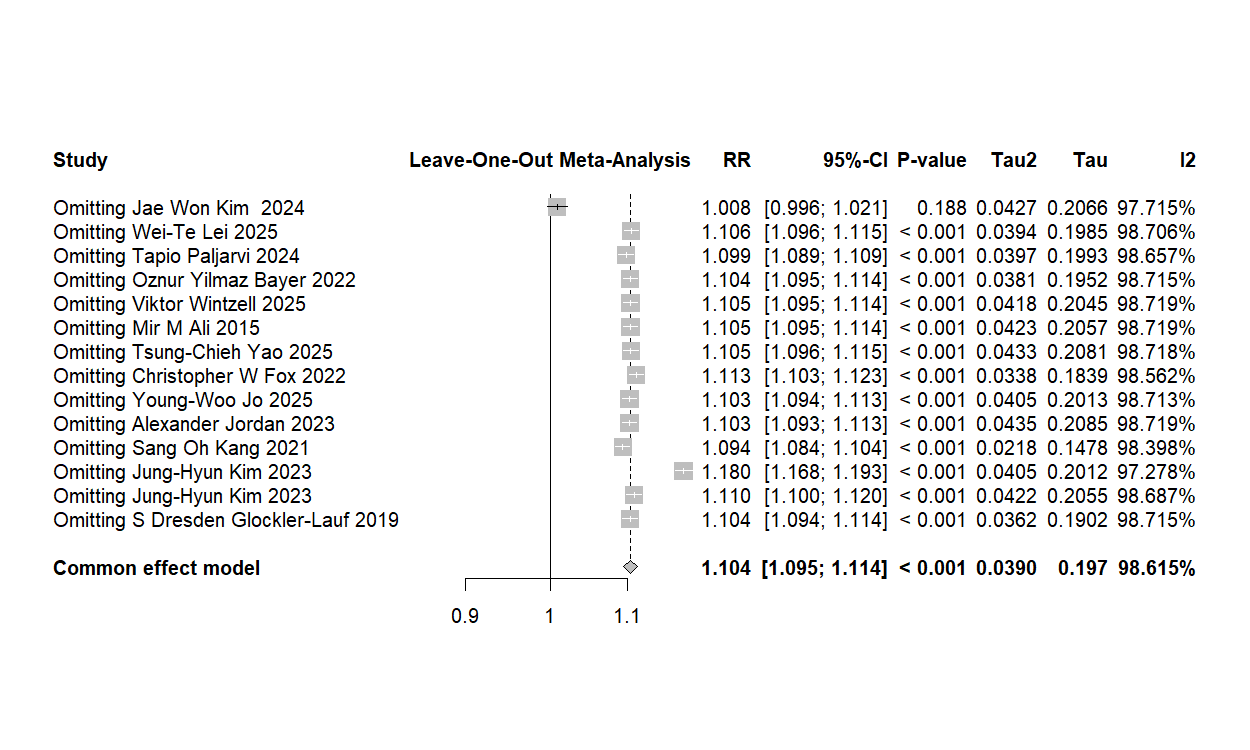

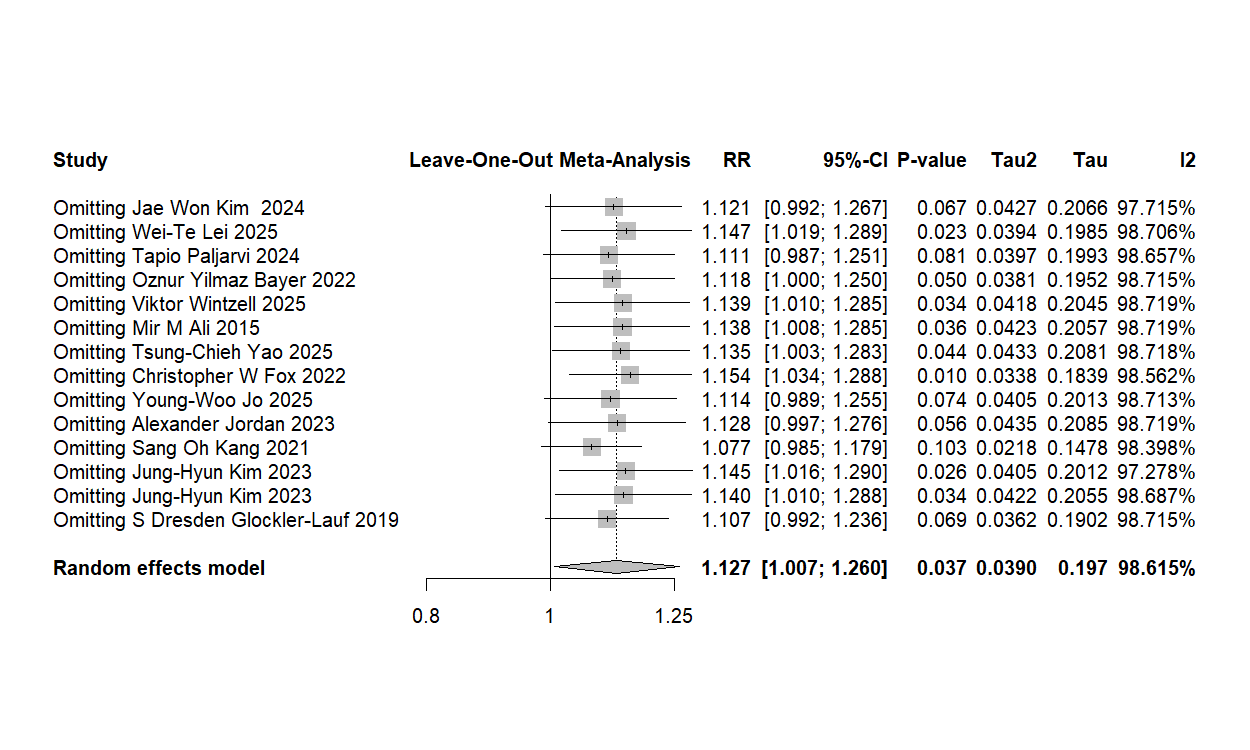


(Left: Common effect model; Right: Random effects model)

RR: Risk Ratio; CI: Confidence interval. The pooled estimate remained robust in most leave-one-out analyses. However, the exclusion of Jae Won Kim 2024 resulted in a loss of statistical significance under the random-effects model (RR = 1.121, 95% CI 0.992–1.267, p=0.067), indicating that this study contributed substantially to the overall significance. Conversely, omitting Christopher W Fox 2022 strengthened the association (RR = 1.154, 95% CI 1.034–1.288, p=0.010). The overall consistency across analyses confirms the stability of the primary conclusion.

eFigure 2: Sensitivity analysis between Suicide and LTRA.


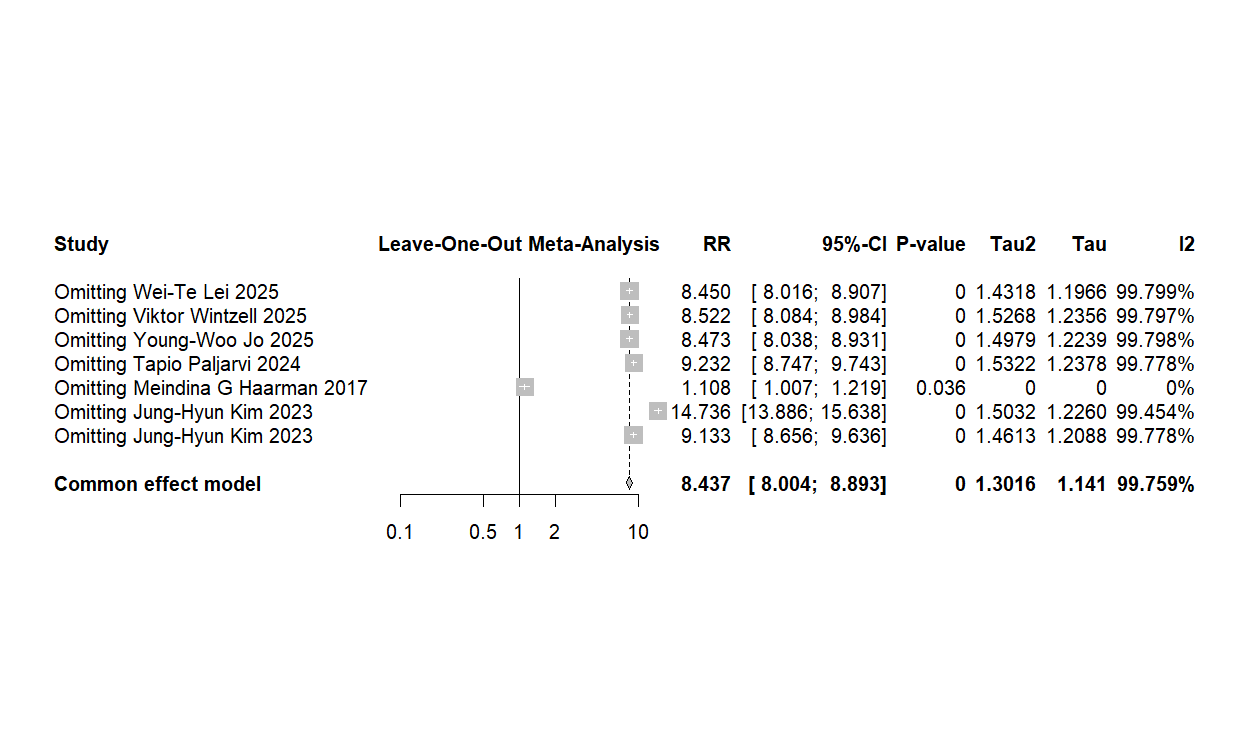

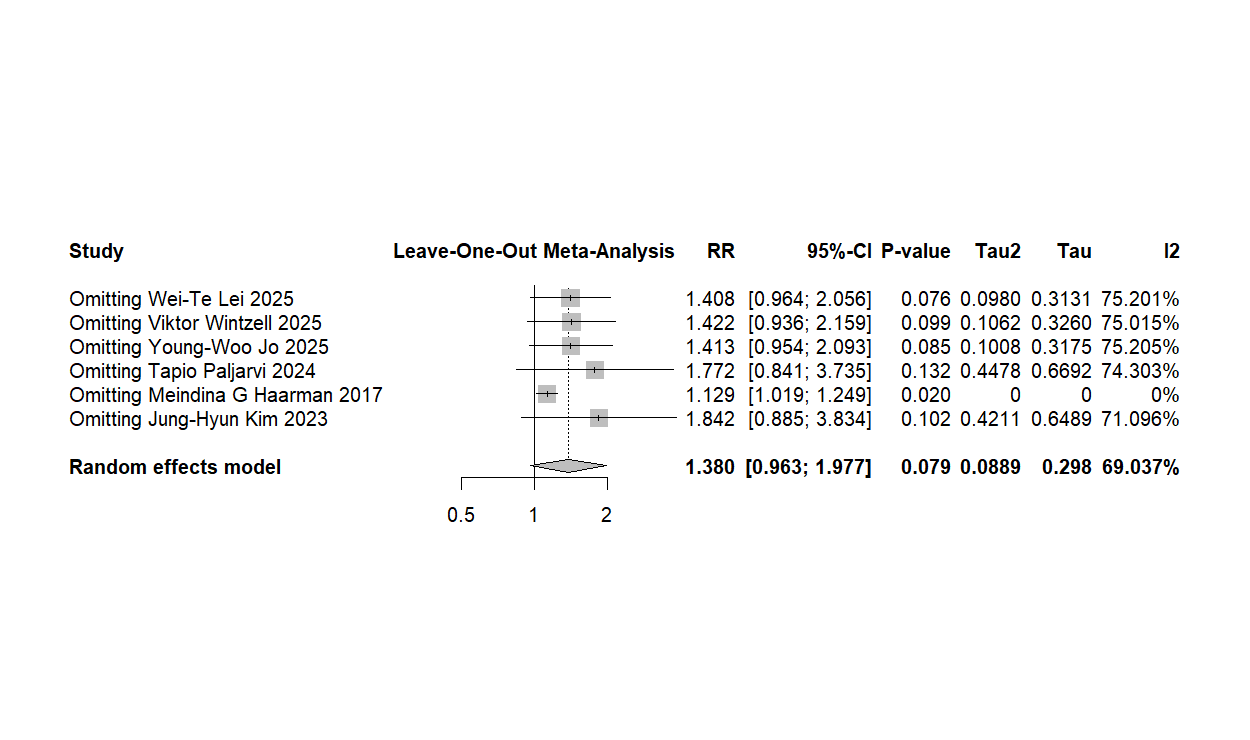


(Left: Common effect model; Right: Random effects model)

RR: Risk Ratio; CI: Confidence interval. The leave-one-out analysis revealed substantial instability in the pooled estimates, which can be attributed in part to the limited number of included studies. The common effect model was heavily influenced by Meindina G Haarman 2017, which alone dramatically attenuated the association (RR = 1.108 vs. overall RR = 8.437). In the random effects model, the overall association was non-significant (RR = 1.380, 95% CI 0.963-1.977, p=0.079), and only the exclusion of Meindina G Haarman 2017 yielded a statistically significant result (RR = 1.129, 95% CI 1.019-1.249, p=0.020). These findings indicate that the observed association is highly dependent on a single study and should be interpreted with caution, particularly given the small number of studies available for this analysis.

eFigure 3: Sensitivity analysis between Psychosis and LTRA.


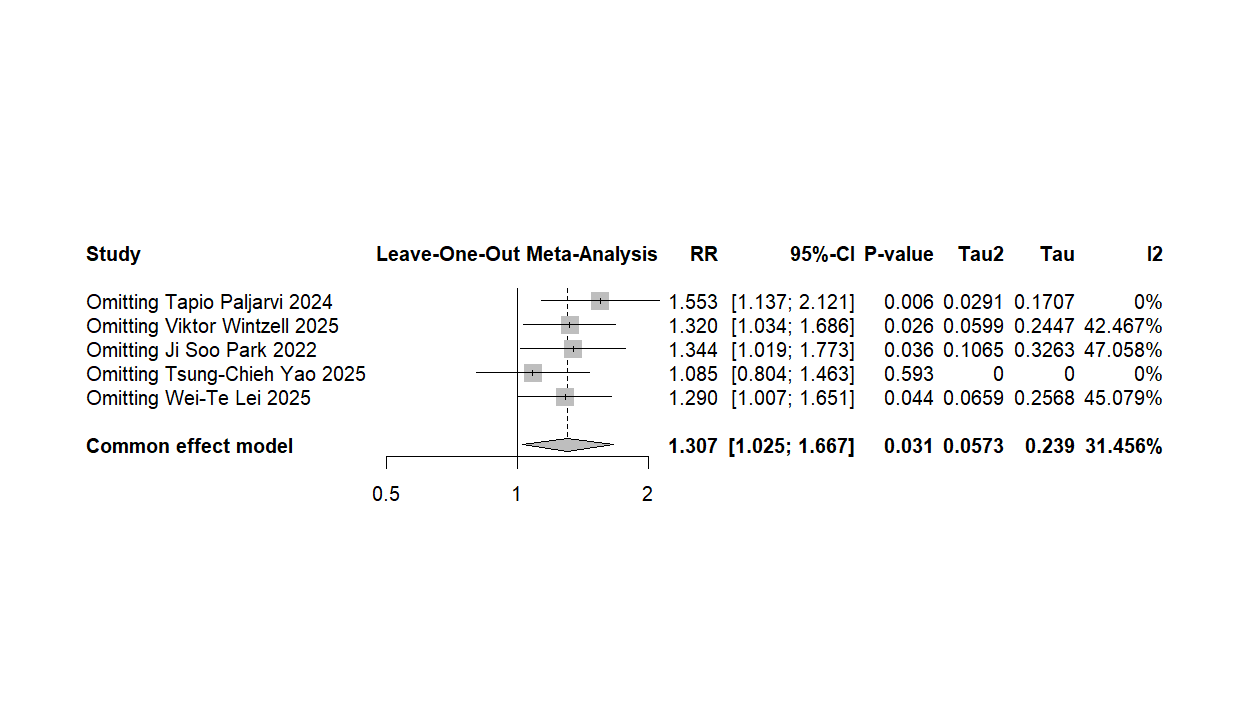

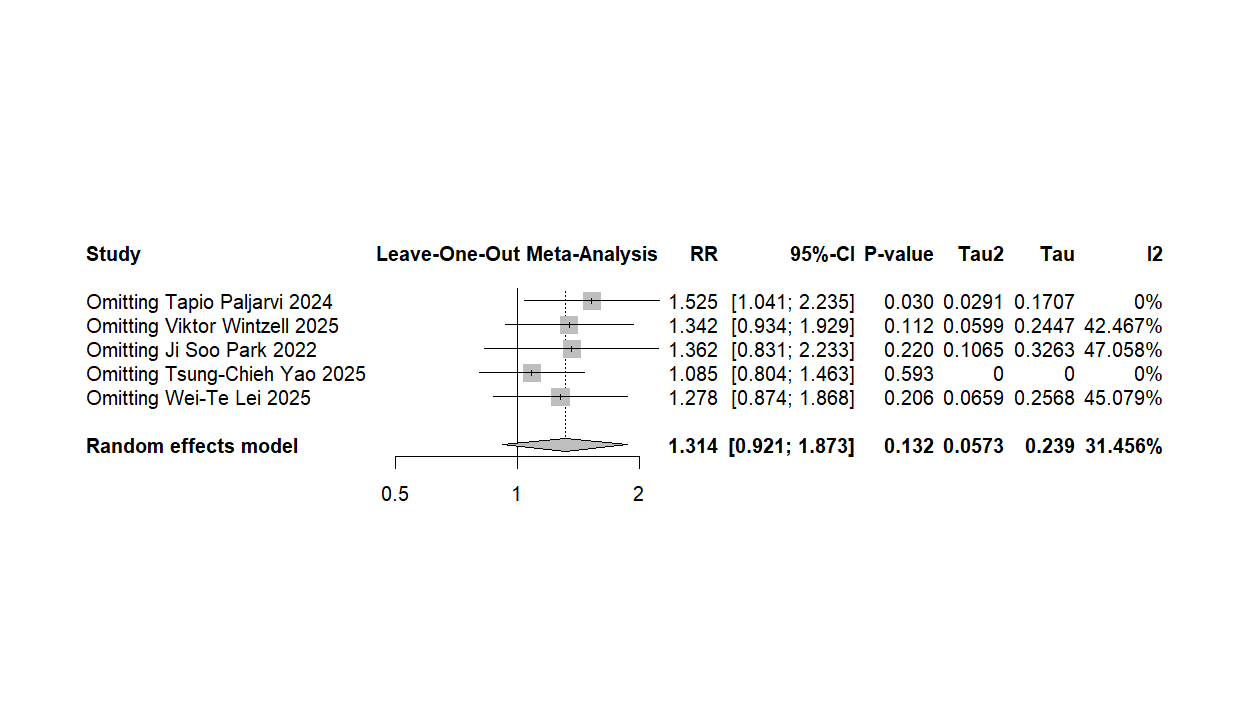


(Left: Common effect model; Right: Random effects model)

RR: Risk Ratio; CI: Confidence interval. The stability of the association varied significantly across sensitivity analyses. In the common effect model, the finding remained statistically significant in four of the five leave-one-out analyses. However, in the random effects model, the association became non-significant in all but one analysis. The substantial fluctuation in effect size and statistical significance upon omitting specific studies (e.g., Tsung-Chieh Yao 2025) indicates limited robustness. This instability is likely attributable to the inherent clinical and methodological heterogeneity among the limited number of included studies, such as variations in study design, population characteristics, and psychosis definition, which could not be fully reconciled despite rigorous methodology.

eFigure 4: Sensitivity analysis between Mood Disorders and LTRA.


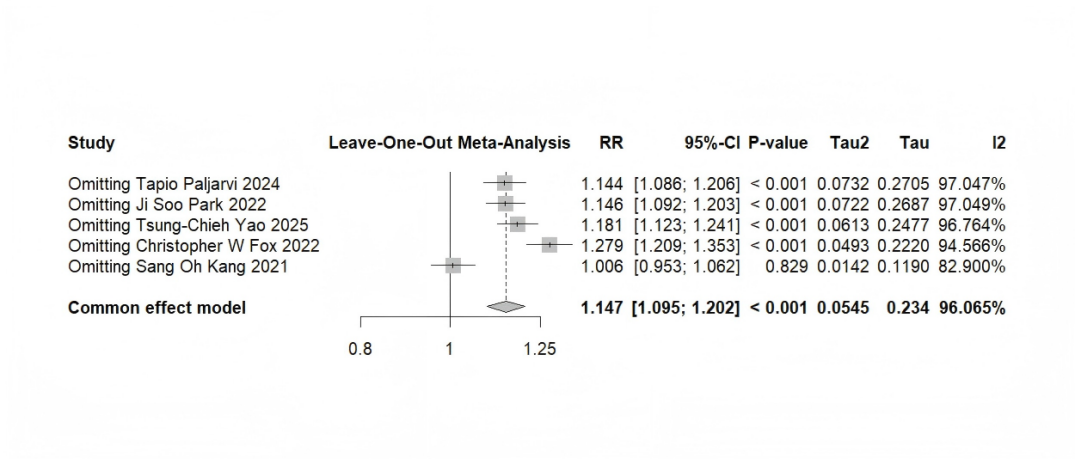

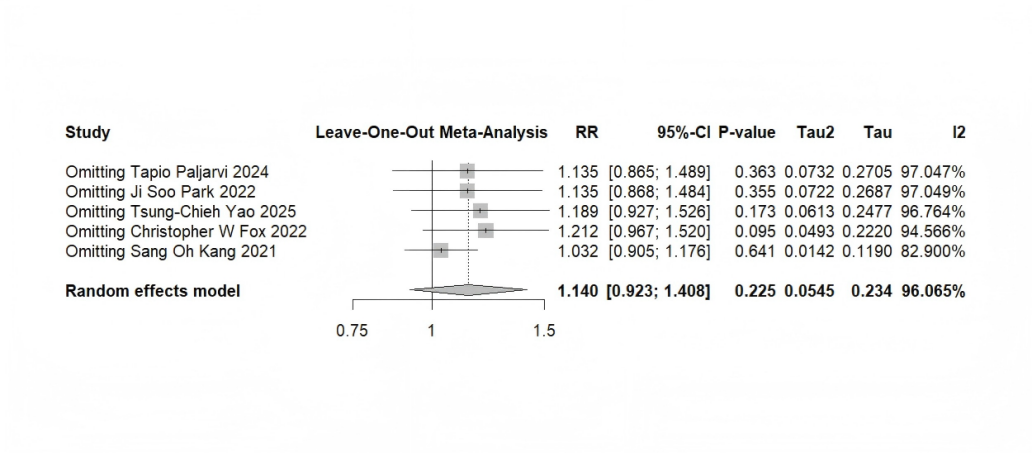


(Left: Common effect model; Right: Random effects model)

RR: Risk Ratio; CI: Confidence interval. The pooled association showed significant instability across models. In the common effect model, the result remained statistically significant in most leave-one-out analyses. However, in the more conservative random effects model, all results became non-significant. The exclusion of Sang Oh Kang 2021 in the common effect model completely attenuated the association to null (RR = 1.006, 95% CI 0.953-1.062, p=0.829), while the exclusion of Christopher W Fox 2022 yielded the strongest association (RR = 1.279). This marked model dependency and study-specific influence likely stem from the combination of a limited number of primary studies and profound underlying heterogeneity (I² > 94%), which may reflect variations in study design, mood disorder definitions, patient populations, or adjustment for confounding factors across the included studies.

eFigure 5: Sensitivity analysis between Sleep Disorders and LTRA.


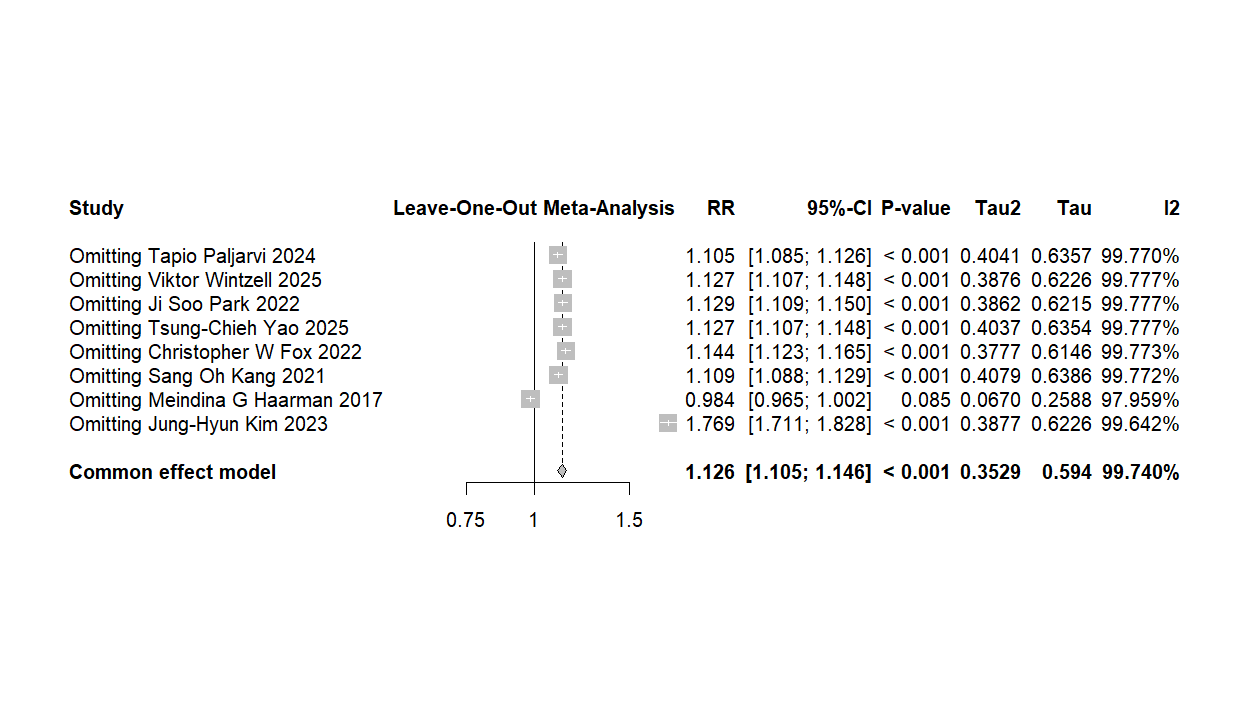

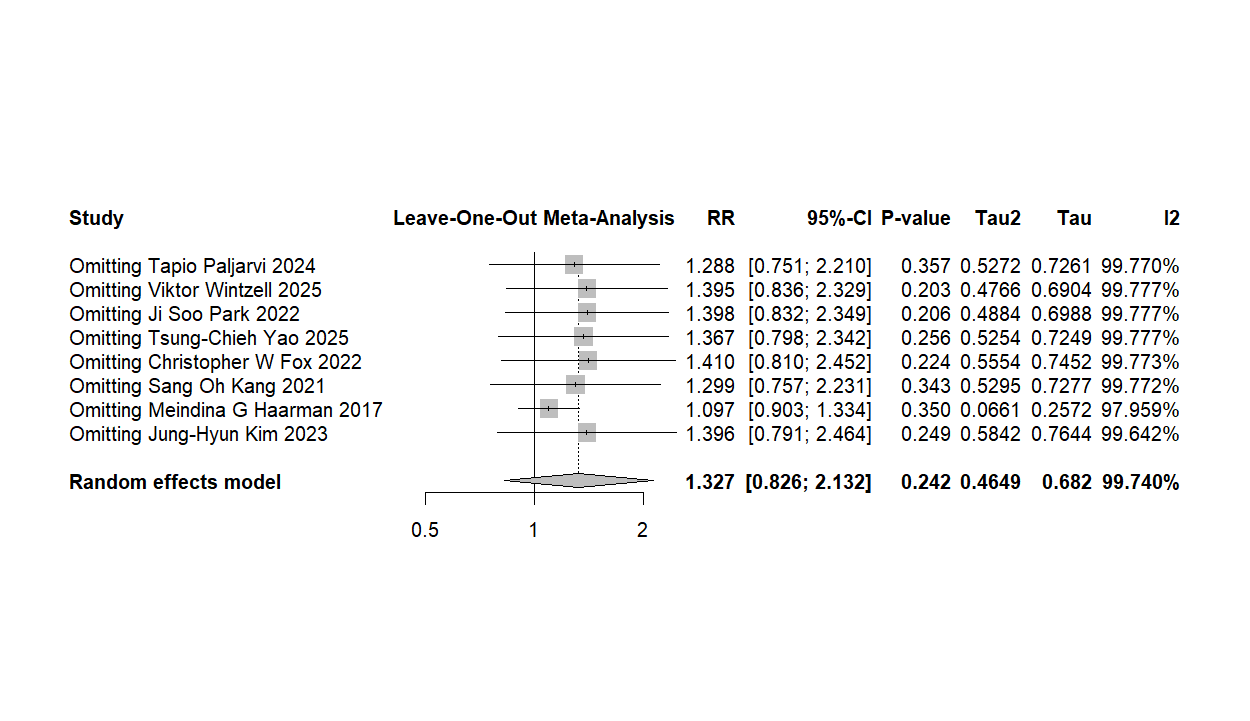


(Left: Common effect model; Right: Random effects model)

RR: Risk Ratio; CI: Confidence interval. Results show marked instability. The common effect model yielded significant associations, while the random effects model showed non-significant results. Exclusion of Meindina G Haarman 2017 nullified the association, while excluding Jung-Hyun Kim 2023 substantially increased effect size. The extreme heterogeneity (I² > 99.7%) and substantial influence of individual studies indicate limited robustness, likely due to clinical and methodological variations across studies that cannot be resolved analytically.

eFigure 6: Sensitivity analysis between Depression and LTRA.


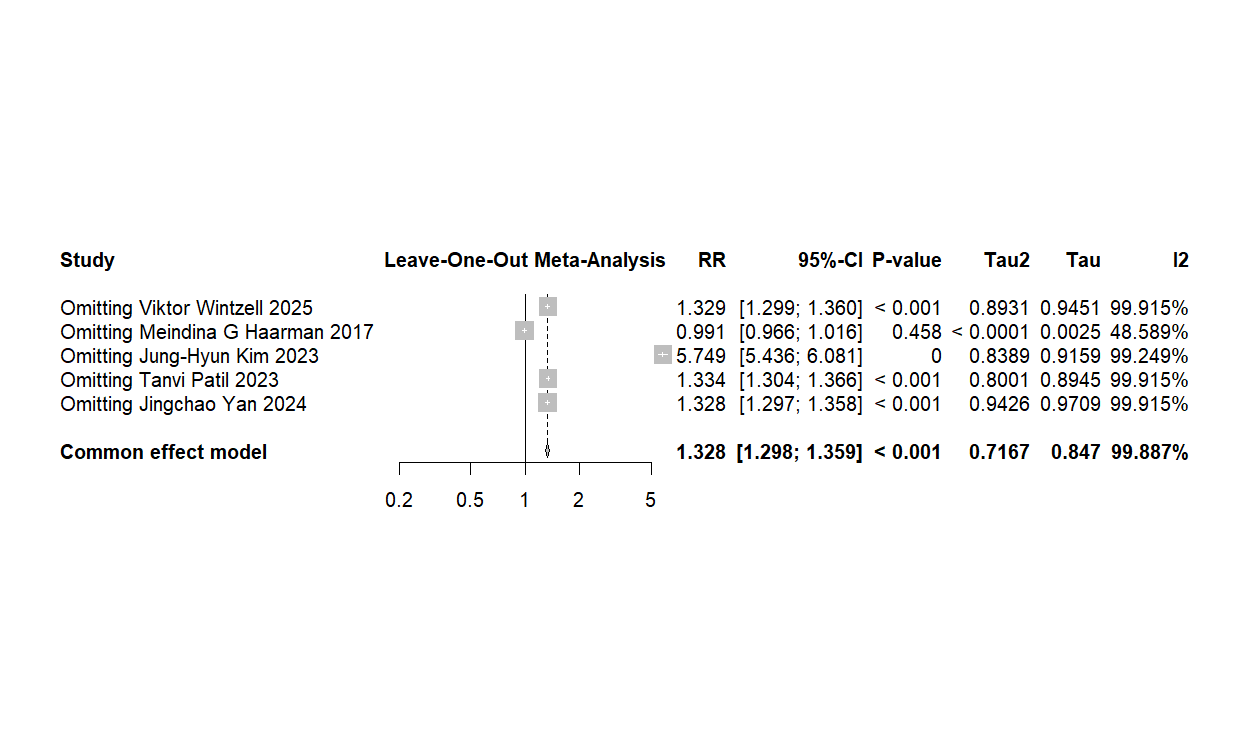


The sensitivity analysis indicated that the significance of the pooled outcome relied heavily on the Jung-Hyun Kim (2023) study. Owing to extreme heterogeneity (I² = 99.9%), the random-effects model was unstable. Specifically, removing the high-weight study led the outlier (Meindina G Haarman, 2017) to dominate the τ² estimation, causing substantial volatility in the pooled estimate and making a meaningful random-effects sensitivity plot unfeasible.

eFigure 7: Publication bias and Egger test on Anxiety.


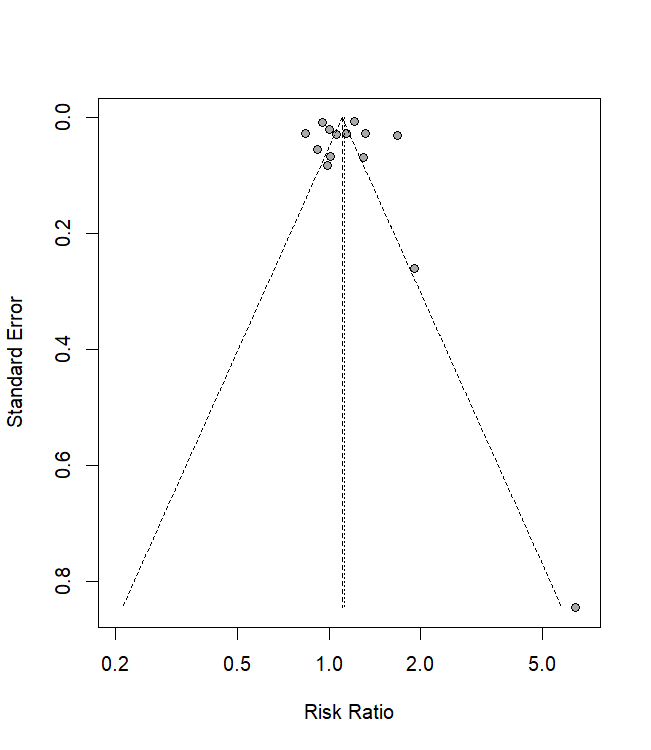

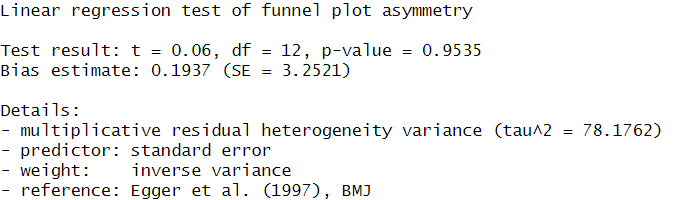

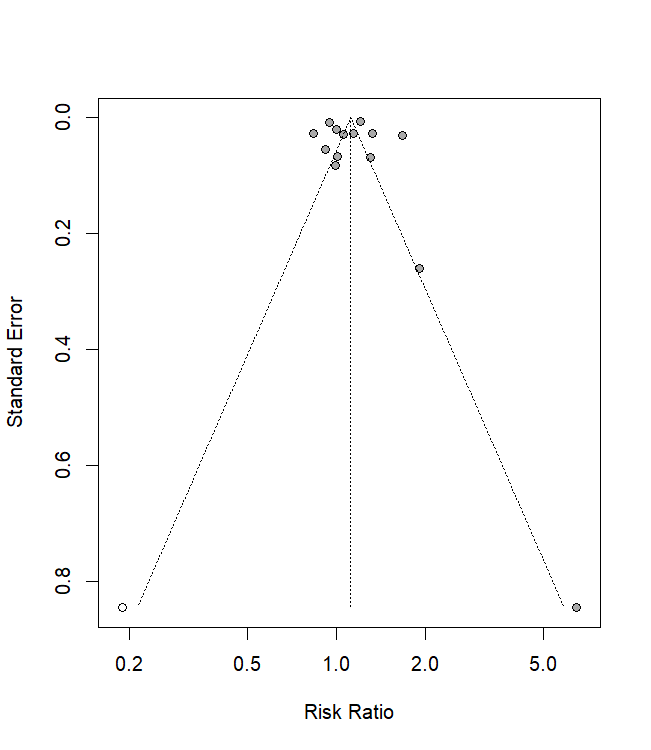


The funnel plots demonstrate a mildly asymmetrical distribution of effect estimates. However, Egger's test shows no statistically significant publication bias (t = 0.06, df = 12, p = 0.9535). The observed mild asymmetry may be related to the moderate heterogeneity (tau² = 78.1762) among studies rather than true publication bias.

eFigure 8: Publication bias and Egger test on Suicide.


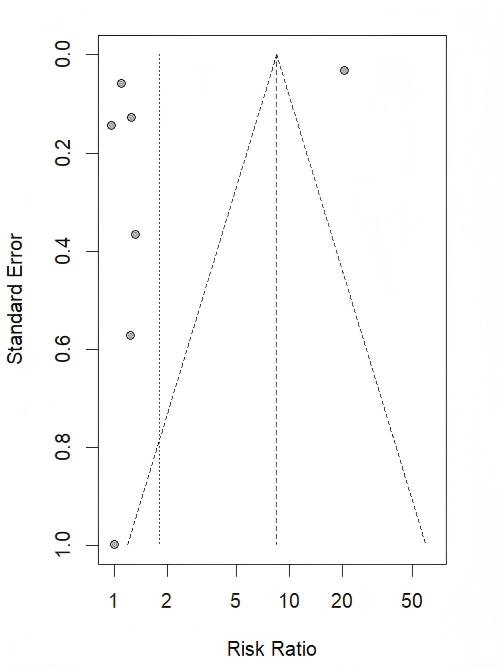

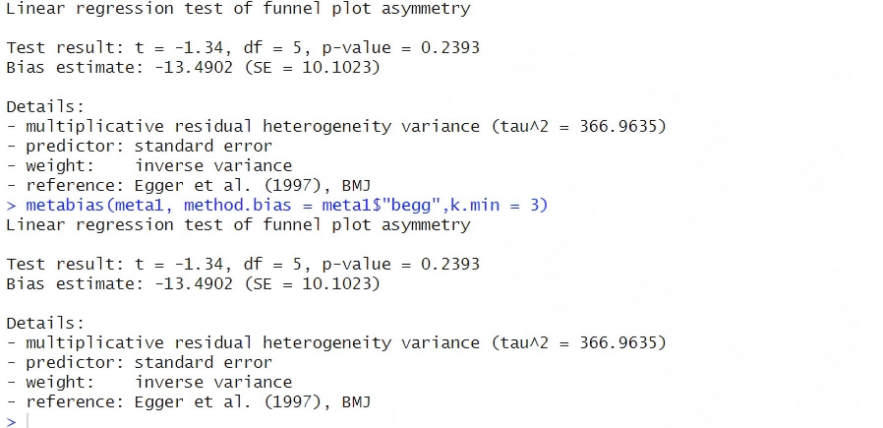

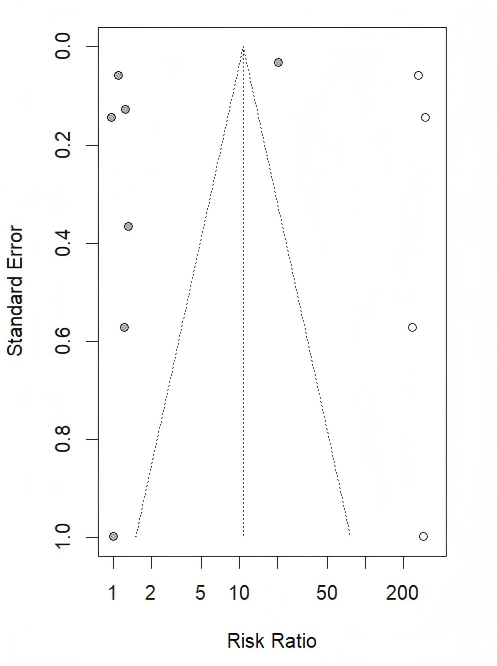


The funnel plot shows an asymmetrical distribution of effect estimates, with considerable scatter among studies. Egger's test indicates no statistically significant publication bias (t = -1.34, df = 5, p = 0.2393). The observed asymmetry is more likely attributable to the substantial heterogeneity (tau² = 366.9635) and clinical diversity among studies rather than publication bias.

eFigure 9: Publication bias and Egger test on Psychosis.


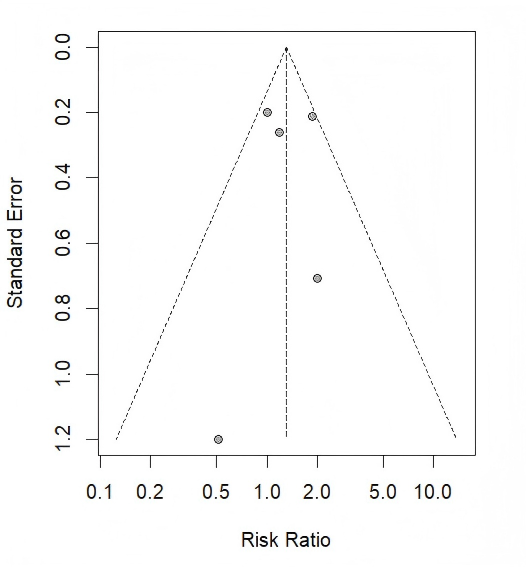

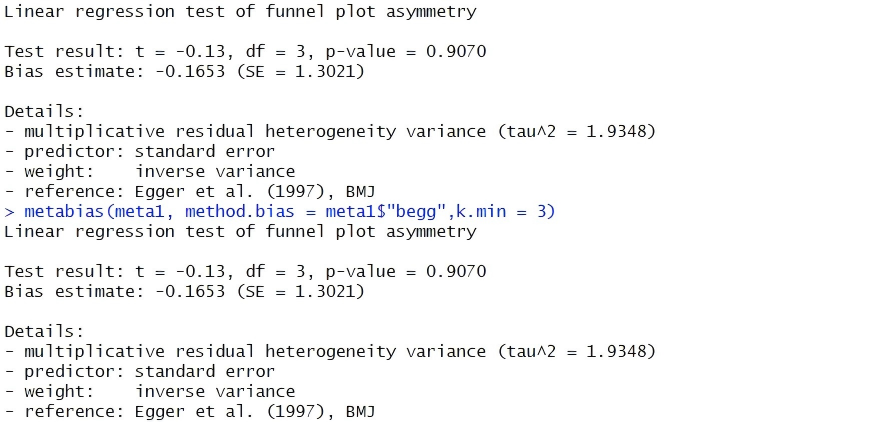


The funnel plot shows a roughly symmetrical distribution of effect estimates around the pooled mean. Egger's test confirms no significant publication bias (t = -0.13, df = 3, p = 0.9070). These findings suggest that the meta-analysis results for psychosis are unlikely to be substantially influenced by publication bias.

eFigure 10: Publication bias and Egger test on Mood Disorders.


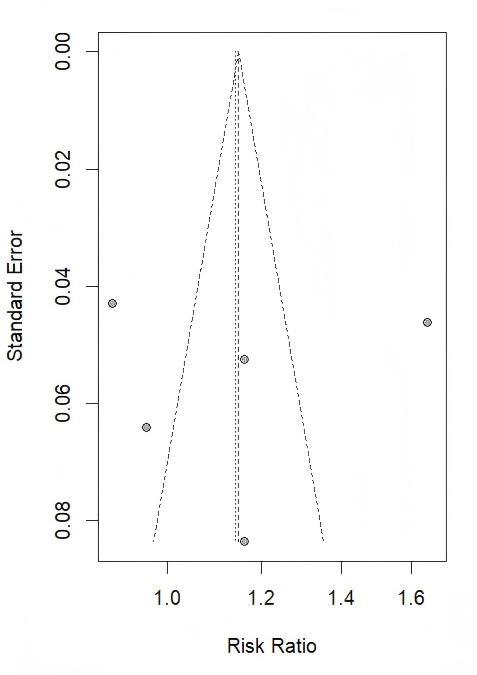

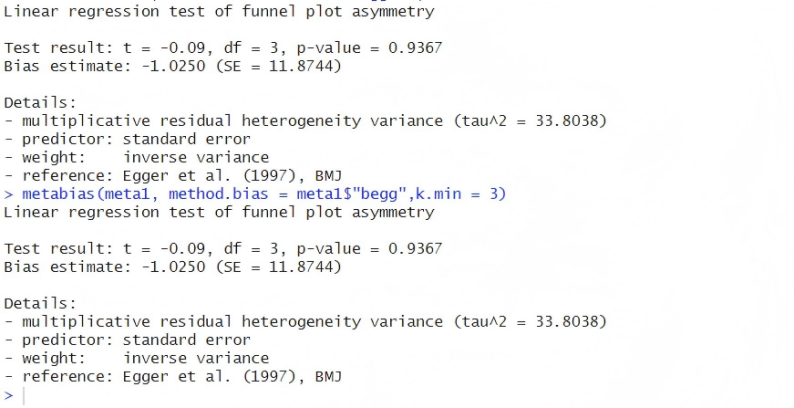

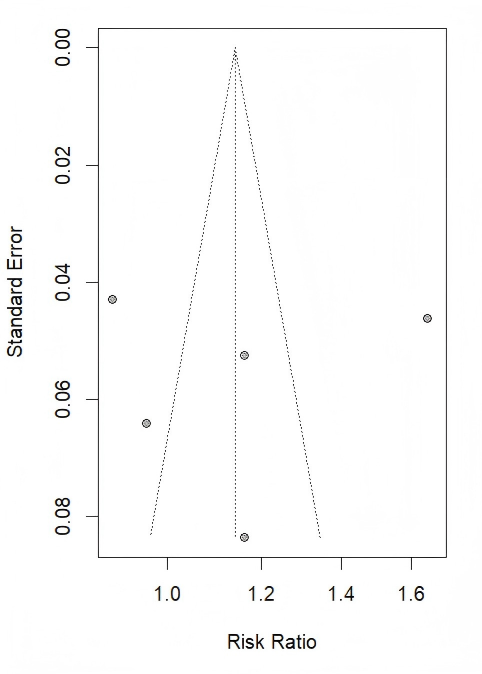


The funnel plot shows an asymmetrical distribution of effect estimates. However, Egger's test indicates no statistically significant publication bias (t = -0.09, df = 3, p = 0.9367). The observed asymmetry may be related to the limited number of studies and substantial heterogeneity (tau² = 33.8038) rather than true publication bias.

eFigure 11: Publication bias and Egger test on Sleep Disorders.


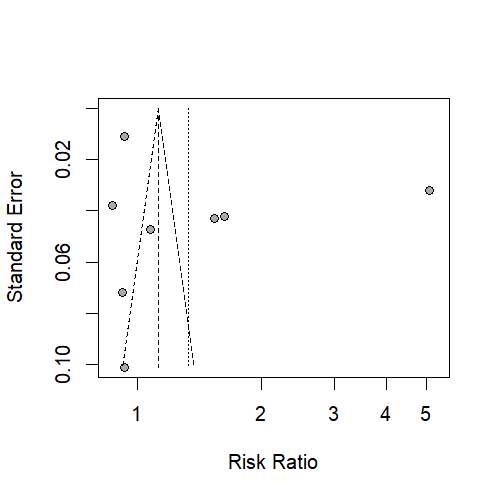

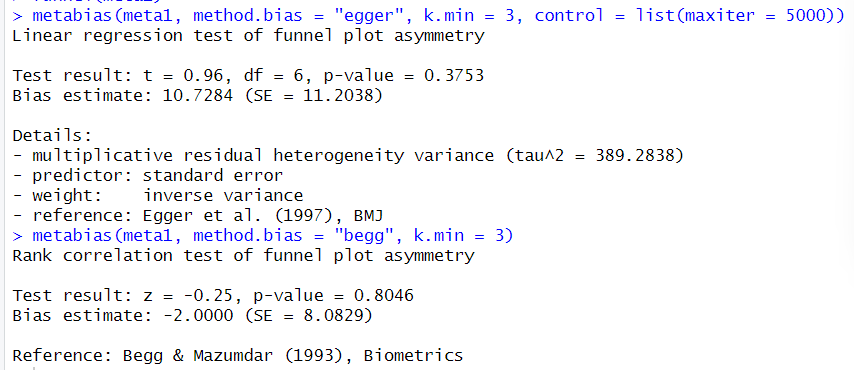

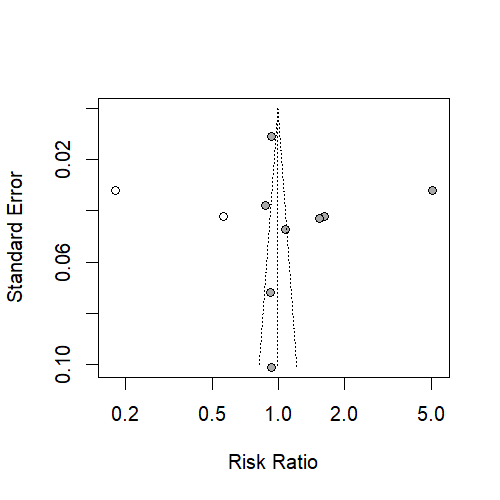


The funnel plots show an asymmetrical distribution of effect estimates, with considerable scatter among studies. Egger's test (t = 0.96, df-6, p = 0.3753) shows no statistical evidence of publication bias. However, the substantial heterogeneity (tau² = 389.2838) and visual asymmetry suggest that the observed dispersion is more likely attributable to clinical and methodological diversity among the limited number of studies rather than publication bias.

eFigure 12: Publication bias and Egger test on Depression.


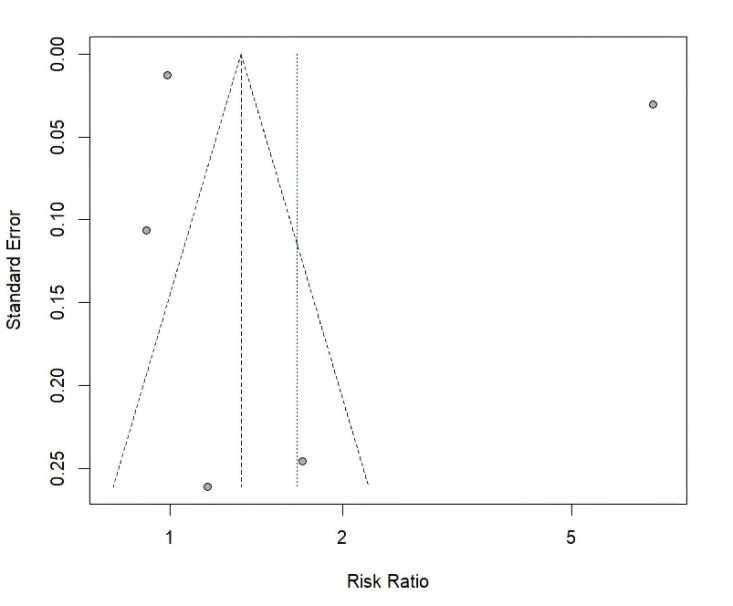

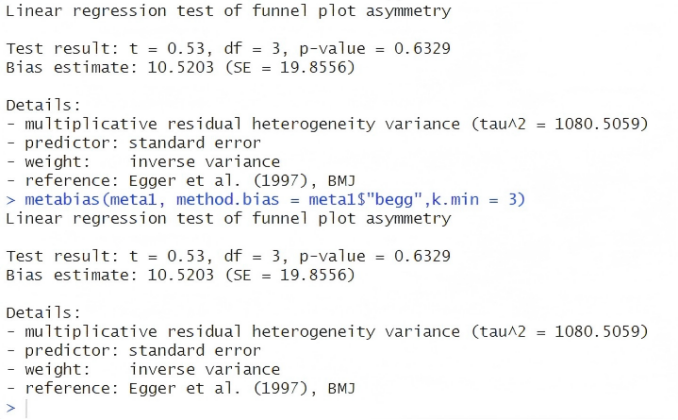

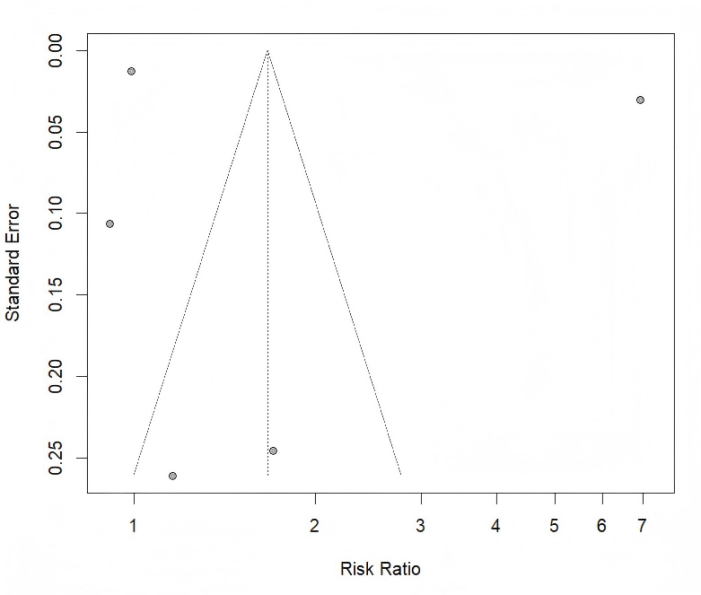


The funnel plots show an asymmetrical distribution of effect estimates with substantial scatter. Egger's test indicates no significant publication bias (t = 0.53, df = 3, p = 0.6329). The observed asymmetry is more likely attributable to the extreme heterogeneity (tau² = 1080.5059) and clinical diversity among the limited number of studies rather than publication bias.

#

# eTable 1. List of excluded references and reasons for exclusion.

| **Title** | **Author** | **Exclusion of cause** |
| --- | --- | --- |
| Neuropsychiatric reactions with the use of montelukast | Corine Ekhart 2022 | Case reprot |
| Effect of concomitant use of montelukast and efavirenz on neuropsychiatric adverse events | [Olatz Ibarra-Barrueta](https://pubmed.ncbi.nlm.nih.gov/?sort=date&term=Ibarra-Barrueta+O&cauthor_id=24259633) 2014 | Case reprot |
| Study on the risk signal mining related to montelukast in pediatric patients based on the US FDA Adverse Event Reporting System Database | Liu, Y.2023 | Data Deficiency |
| Relationship between montelukast and behavioral problems in preschool children with asthma | Eda Özata 2022 | Data Deficiency |
| Psychiatric Disorders and Montelukast in Children: A Disproportionality Analysis of the VigiBase(®) | Ana Aldea Perona 2016 | Data Deficiency |
| Evaluation of Neuropsychiatric Effects of Montelukast-Levocetirizine Combination Therapy in Children with Asthma and Allergic Rhinitis | Uğur Alta 2023 | Data Deficiency |
| Neuropsychiatric Adverse Events of Montelukast: An Analysis of Real-World Datasets and drug-gene Interaction Network | Ryogo Umetsu 2021 | Data Irrelevance |
| Advancing Evidence for Neuropsychiatric Disorders (Depression, Anxiety, and Sleep) With Leukotriene Receptor Antagonists in Selected Patients | Larenas-Linnemann, D.E.S. 2025 | Editorial |
| Anti-allergic compounds in chronic pelvic pain | Theoharides, T.C 2017 | Full Text Missing |
| Risk of Neuropsychiatric Diseases According to the Use of a Leukotriene Receptor Antagonist in Middle-Aged and Older Adults with Asthma: A Nationwide Population-Based Study Using Health Claims Data in Korea | Ji-Su Shim 2021 | Full Text Missing |
| Meta-analysis of the relationship between montelukast use and neuropsychiatric events in patients with allergic airway disease | [Yakui Mou](https://pubmed.ncbi.nlm.nih.gov/?sort=date&term=Mou+Y&cauthor_id=38034763) 2023 | Randomized controlled trail |
| Montelukast for children with obstructive sleep apnea: a double-blind, placebo-controlled study | Aviv D Goldbart 2012 | Randomized controlled trail |

**eTable 2. NEWCASTLE - OTTAWA QUALITY ASSESSMENT SCALE (COHORT STUDIES)**

| Author, year | Selection | | | | Comparability | Outcome | | | Overall  quality |
| --- | --- | --- | --- | --- | --- | --- | --- | --- | --- |
|  | Representative of cohort | Selection of cohort | Exposure ascertainment | No history of disease | Comparability of cohorts | Outcome assessment | Follow-up long enough(median ≥ 5 years) | Adequacy of follow up |  |
| Christopher W Fox 2022 | 1 | 1 | 1 | 1 | 1 | 1 | 0 | 0 | 6 |
| S Dresden Glockler-Lauf 2019 | 1 | 1 | 1 | 1 | 2 | 1 | 0 | 0 | 7 |
| Po-Yu Huang 2021 | 1 | 1 | 1 | 1 | 2 | 1 | 0 | 0 | 7 |
| Wei-Te Lei 2025 | 1 | 1 | 1 | 1 | 2 | 1 | 0 | 0 | 7 |
| [Min-Lan Tsai 2022](https://pubmed.ncbi.nlm.nih.gov/?sort=date&term=Tsai+ML&cauthor_id=36360335) | 1 | 1 | 1 | 1 | 2 | 1 | 0 | 0 | 7 |
| Hui-Ju Tsai 2023 | 1 | 1 | 1 | 0 | 2 | 1 | 0 | 0 | 6 |
| Tsung-Chieh Yao 2025 | 1 | 1 | 1 | 1 | 2 | 1 | 0 | 0 | 7 |
| Alexander Jordan 2023 | 1 | 1 | 1 | 1 | 1 | 1 | 0 | 0 | 6 |
| Meindina G Haarman 2017 | 1 | 1 | 1 | 0 | 0 | 1 | 0 | 0 | 4 |
| Young-Woo Jo 2025 | 1 | 1 | 1 | 1 | 2 | 1 | 0 | 0 | 7 |
| Sang Oh Kang 2021 | 1 | 1 | 1 | 1 | 2 | 1 | 0 | 0 | 7 |
| Ji Soo Park 2022 | 1 | 1 | 1 | 1 | 1 | 1 | 0 | 0 | 6 |
| Jung-Hyun Kim 2023 | 1 | 1 | 1 | 1 | 2 | 1 | 0 | 0 | 7 |
| Jae Won Kim 2024 | 1 | 1 | 1 | 1 | 2 | 1 | 0 | 0 | 7 |
| Viktor Wintzell 2025 | 1 | 1 | 1 | 1 | 2 | 1 | 0 | 0 | 7 |
| Oznur Yilmaz Bayer 2022 | 1 | 0 | 1 | 1 | 0 | 1 | 0 | 1 | 5 |
| Mir M Ali 2015 | 1 | 1 | 1 | 1 | 2 | 1 | 0 | 0 | 7 |
| Jingchao Yan 2024 | 1 | 1 | 0 | 1 | 2 | 1 | 0 | 0 | 6 |
| Ducharme, F.M 2017 | 1 | 1 | 1 | 1 | 1 | 1 | 0 | 0 | 6 |
| Tapio Paljarvi 2024 | 1 | 1 | 1 | 1 | 2 | 1 | 0 | 0 | 7 |
| Tanvi Patil 2023 | 1 | 1 | 1 | 1 | 2 | 1 | 0 | 0 | 7 |

**eTable 3. Grade Grading Details**

| **Outcomes** | **Relative effect difference (95% CI)** | **Absolute effect difference (95% CI** | **No of Participants (studies)** | **Quality of the evidence (GRADE)** |
| --- | --- | --- | --- | --- |
| All | RR=1.11,[95%CI 0.98,1.26] | - | 2199309 | ⨁⨁◯◯ |
|  |  |  | （16） | Low (b-,c+) |
| Anxiety | RR=1.25,[95%CI 0.86,1.82] | - | 770421 | ⨁◯◯◯ |
|  |  |  | (9) | Very low (b-) |
| Personality disorder | RR=0.99,[95%CI 0.58,1.69] | - | 275354 | ⨁⨁◯◯ |
|  |  |  | (3) | Low |
| Suicide | RR=2.02,[95%CI 0.72,5.66] | - | 597914 | ⨁◯◯◯ |
|  |  |  | (6) | Very low (b-,d-,e-,a+) |
| Psychosis | RR=1.31,[95%CI 0.92,1.87] | - | 457029 | ⨁⨁◯◯ |
|  |  |  | (5) | Low |
| Mood Disorders | RR=1.41,[95%CI 0.92,1.41] | - | 570599 | ⨁◯◯◯ |
|  |  |  | (5) | Very low (b-,e-) |
| Sleep Disorders | RR=1.33,[95%CI 0.88,2.01] | - | 755833 | ⨁◯◯◯ |
|  |  |  | (8) | Very low (b-,e-) |
| Depression | RR=1.66,[95%CI 0.78,3.54] | - | 302032 | ⨁◯◯◯ |
|  |  |  | (5) | Very low (b-,e-) |
| Emotional disorder | RR=1.03,[95%CI 0.68,1.57] | - | 258353 | ⨁◯◯◯ |
|  |  |  | (2) | Very low (b-) |
| Autism | RR=1.89,[95%CI 0.62,1.29] | - | 590745 | ⨁⨁◯◯ |
|  |  |  | (2) | Low |
| Movement | RR=1.20,[95%CI 0.96,1.50] | - | 260766 | ⨁⨁◯◯ |
|  |  |  | (2) | Low |
| ADHD | RR=0.99,[95%CI 0.87,1.12] | - | 645232 | ⨁⨁◯◯ |
|  |  |  | (3) | Low |
| Tourette syndrome | RR=1.36,[95%CI 1.23,1.50] | - | 644713 | ⨁⨁◯◯ |
|  |  |  | (3) | Low |

Note:

a-: Risk of bias in the study; b-: Inconsistencies between studies; c-: indirectness; d-: Accuracy of the findings; e-: Publication bias or small sample size

a+: Based on consistent evidence from two or more observational studies with no known confounders, RR>2(<0.5) (+1); b+: Direct evidence based on real and no

significant threat RR>2(<0.5) (+2); c+: Evidence of dose-response gradient (+1); d+: All known confounders reduced the effect (+1).

Cohort studies are usually evaluated from a low level (⨁⨁◯◯).

⨁⨁⨁◯: Evidence of moderate quality suggests that the current efficacy evaluation results are likely to be close to the true value;

⨁⨁◯◯: Low quality research evidence suggests that the reliability of current efficacy evaluation results is uncertain;

⨁◯◯◯: Very low quality research evidence, suggesting uncertainty about the reliability of current efficacy evaluation results;
